# A Comprehensive Survey on MIMO Visible Light Communication: Current Research, Machine Learning and Future Trends

**DOI:** 10.3390/s23020739

**Published:** 2023-01-09

**Authors:** Mohammad Abrar Shakil Sejan, Md Habibur Rahman, Md Abdul Aziz, Dong-Sun Kim, Young-Hwan You, Hyoung-Kyu Song

**Affiliations:** 1 Department of Information and Communication Engineering, Sejong University, Seoul 05006, Republic of Korea; 2 Department of Convergence Engineering for Intelligent Drone, Sejong University, Seoul 05006, Republic of Korea; 3 Department of Electrical Engineering, Sejong University, Seoul 05006, Republic of Korea; 4 Department of Computer Engineering, Sejong University, Seoul 05006, Republic of Korea

**Keywords:** MIMO, machine learning, VLC, wireless communication, optical communication

## Abstract

Visible light communication (VLC) has contributed new unused spectrum in addition to the traditional radio frequency communication and can play a significant role in wireless communication. The adaptation of VLC technology enhances wireless connectivity both in indoor and outdoor environments. Multiple-input multiple-output (MIMO) communication has been an efficient technique for increasing wireless communications system capacity and performance. With the advantages of MIMO techniques, VLC can achieve an additional degree of freedom. In this paper, we systematically perform a survey of the existing work based on MIMO VLC. We categorize the types of different MIMO techniques, and a brief description is given. Different problem-solving approaches are given in the subsequent sections. In addition, machine learning approaches are also discussed in sufficient detail. Finally, we identify the future study direction for MIMO-based communication in VLC.

## 1. Introduction

Wireless communication is now changing at a rapid pace to achieve the design goal of fifth-generation (5G) and beyond 5G (B5G) [1]. The 5G communication network requirements are enhanced mobile broadband, ultra-low latency communication, and massive connectivity [2]. The demand for the increasing number of devices is a great challenge in the current capacity of radio frequency (RF) communication. The previous studies suggested that the majority of the data traffic is generated by indoor users [3]. Thus, the wireless service will be required in indoor environments more as compared to outdoor considering bandwidth usage in both industrial and general households. In the upcoming days, the demand for internet access will have exponential growth as two-thirds of the world’s population will be connected to the internet [4]. Thus, new communication technologies and bandwidth are required to enhance the user experience and ensure connectivity.

Visible light communication (VLC) is a technology for wireless communication that uses light signals to transfer data to the receiving device [5]. VLC exhibits a great feature of illumination and communication at the same time. The visible light spectrum has a large bandwidth which can be an additional solution for radio frequency (RF) communication. The visible light spectrum ranges from 380 nm to 750 nm, corresponding to a frequency spectrum in the range of 430 THz to 790 THz [6]. The spectrum scarcity in RF can impose limitations on device connectivities of the internet of things (IoT) where VLC can provide a promising solution [7,8,9]. In addition, VLC provides high bandwidth density (b/s/m${}^{2}$) stemming from the optical signal and broad adaptation of lighting infrastructure indoors [10]. VLC has the advantages like unlicensed and large unused bandwidth, and security is high because the light signal cannot pass through walls. The transmitter and receiver are cheap, so the implementation cost is less. Light emitting diodes (LEDs) are used as transmitters, and photodiodes or complementary metal–oxide–semiconductor (CMOS) cameras can utilize as receivers [11,12]. LEDs contain some advantages like long life, cheap manufacturing cost, and wide adaptation in indoor illumination [13,14]. VLC has a lot of research attention in scientific communities. Some of the striking features of VLC can be listed as [15]:

- Large bandwidth is unlicensed and free to use.
- VLC does not interfere with existing RF communication.
- No additional setup is required that the existing illumination system can be used for communication.
- The cost of implementing a VLC-based transmitter and receiver is less compared to the RF system.
- Illumination and Communication are possible at the same time.
- The health risk does not exist for humans apart from the flickering effect, which can be mitigated by using a modulation frequency of more than 200 Hz.
- As the receiver size is small, multipath fading can be mitigated.

**Figure 1:**
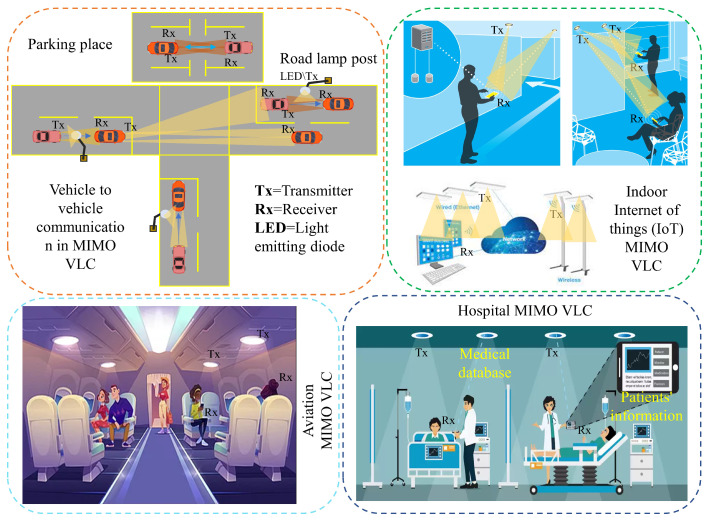
Overview of the VLC-based MIMO communication applications.

Multiple-input multiple-output (MIMO) uses multiple antennas in the transmitters and receivers instead of one single antenna. MIMO communication helps to increase channel capacity substantially and can ensure higher data throughput [16]. Other benefits of MIMO are the use of inexpensive low-power components, reduced latency, simplified medium access control (MAC) layer, and robustness against jamming [17]. In addition, multiple users can be supported in an efficient way. A promising solution to boost the data rate without any bandwidth or power expansion is achieved by using MIMO techniques [18]. MIMO communication in VLC has also been studied in a comprehensive manner. Both simulation and experimental studies were performed to demonstrate the advantage of MIMO communication. An overview of application scenarios is given in Figure 1 utilizing the MIMO VLC system. Different examples can be made by using MIMO and VLC to enhance communication performance.

However, there are several challenges that exist in MIMO VLC, and the scientific community is actively researching to find perfect solutions. Rising co-channel interference (CCI) noise brought on by many LEDs at the transmitters and receivers, respectively, is one of the difficulties in adopting MIMO systems [19]. Crosstalk between the LED transmitters is the reason for CCI occurrence. The constant modulus algorithm (CMA) application might be used to resolve this problem. CMA is referred to as blind adaptive equalization that makes use of the signal’s underlying constant modulus feature [20]. Compared to the simplified constant modulus algorithm (SCMA) and modified constant modulus algorithm (MCMA), which both employ restricted phase information, the CMA for the MIMO setup is more robust to phase noise [21]. Even though CMA may be employed to lower CCI [22], the carrier frequency offset is a problem. The kHz range of MCMA and SCMA can be used to correct this offset. Recently, ref. [23] proposed a constrained field-of-view angular diversity receiver (CFOV-ADR) which successfully reduces the CCI. The NLOS signal, however, was regarded as an interfering signal in the investigation.

### 1.1. Related Literature

In the previous literature, a good amount of survey papers have been published focusing on VLC. We describe the related works in chronological order of the papers for VLC. Smart lighting and free space optical (FSO) were surveyed in [24] published in 2013. The study investigated the application of the FSO model and VLC with smart lighting technology. In FSO, two scenarios have been highlighted as stationary scenarios and mobile scenarios. Stationary scenarios are considered as the heaviest usages of FSO as they can provide longer communication ranges and higher data speeds. FSO provides limited mobility, so more investigation needs to be carried out to enhance service for mobile users. Apart from that, future challenges have also been discussed, like upper layer design, solid-state design, mobility, and line-of-sight (LOS) communication. The authors in [13] provided a survey on the VLC system and characteristics, physical layer properties of VLC, medium access techniques, system design and programmable platform, and VLC sensing and applications. The authors also described some of the future implementation issues in building high-capacity mobile VLC networks. Another survey paper was done in [25] and was focused on the advantages of VLC technology over traditional techniques, details of modulation techniques, and methods for improving VLC system performance such as filtering, equalization compensation, and beamforming. The authors also pointed out some of the outstanding limitations of VLC, including uplink connection, interference, shading, lights off mode, effects of LED junction temperature, and challenges in commercialization. Wireless communication can send alternative data traffic using the VLC spectrum, and this opportunity was surveyed in [26]. The authors described VLC advantages, standardization, channel model, VLC receiver types, MAC and network layer description, and multiplexing techniques. The paper also focused on some future potential applications of VLC, like intelligent homes, shopping malls, hospitals, outdoor environments, and underwater communication. Indoor positioning is a challenging task as the global positioning system (GPS) can not provide accurate locations of people or packages. VLC-based technology can be used for tracking or finding locations indoors [27,28]. Indoor positioning application was investigated in [29]. The paper described each related study based on positing algorithms, types of receivers, and multiplexing techniques. Environmental adaptive VLC receivers were focused in [30] for vehicular communication in dynamic traffic situations and in unfriendly atmospheric conditions. The hardware architecture of the VLC receiver was described in the first place for camera-based and photodiode-based receivers. Next, the issues of outdoor communication using VLC and the ways to mitigate those issues were discussed. Finally, the authors proposed a series of adaptive solutions for robust communications. The paper in [7] is a brief description of application scenarios for VLC, architecture, standardization, modulation techniques, and open research issues. The authors in [31] presented different research directions for effective automotive communication using VLC. VLC can be considered for vehicular communication, and challenges regarding VLC usage and future directions were presented in the survey. Again [32] presented an extended study of indoor positioning techniques using VLC. The study categorized positioning algorithms as mathematical methods, sensor-assisted methods, and optimization methods and analyzed the accuracy of the algorithms in experiment and simulation environments. As time progresses, more studies have been added to VLC. In [14], the authors focused on VLC main concepts and research challenges. A description of communication architecture, physical and MAC layers, applications, and challenges were provided. Rehman et al. [33] also surveyed the prospects and challenges at the same time for VLC. The focus was to integrate VLC with RF towards a hybrid communication system for stable communication. The authors in [34] studied the different security threats and vulnerabilities that existed in VLC communication. The authors in [35] covered a survey of the theory of illumination, VLC system receivers, architecture, and ongoing developments. The existing VLC technology can be a potential candidate for 5G, B5G, 6G, and other emerging technologies. To describe the different channel modeling techniques for VLC, a survey was conducted by [36]. The study considered four different channel conditions, including indoor, outdoor, underwater, and underground. Different channel modeling techniques include recursive, iterative, ray-tracing, ceiling bounce, geometric-based stochastic models, Monte Carlo, modified Monte Carlo, LOS channels, geometry-based, measured channels, Beer–Lambert, Random-based, and radiative transfer equations. The advantage and disadvantages were of each channel model technique are also presented. In more recent times, the authors in [37] presented work on integrating VLC technology with the internet of things (IoT), including communication scenarios for machine-to-machine, vehicle-to-infrastructure, infrastructure-to-vehicle, chip-to-chip, and device-to-device. The authors in [38] described key technologies in VLC and application scenarios in VLC, including machine learning approaches. Power line communication can be used as the backbone technology for VLC, and a survey in [39] was conducted.

### 1.2. Motivation and Contributions

MIMO can contribute to additional advantages for VLC in terms of data rate and multiple-user service. However, there is a gap in the literature in conducting a complete survey for the MIMO VLC study. Refs. [13,40] included MIMO as a subsection but were not been extensively studied. A massive MIMO communication survey was performed in [41], which covered both RF and VLC-related studies. Muti-user VLC-based communication was discussed in [42], which included precoding, multiple access, resource allocation, and mobility management. The paper focused on a comprehensive overview of single-user VLC systems, multi-user VLC systems, and future directions. MIMO technology was not discussed in great detail; only user-based MIMO studies were considered. Again, ref. [43] provided a survey on MIMO-orthogonal frequency division multiplexing (OFDM)-based studies in VLC, but, the study did not provide an in-depth analysis of MIMO techniques. Thus, to fill this research gap, we have been motivated to survey MIMO VLC in a comprehensive way. A comparison of different MIMO VLC studies is listed in Table 1. Our contributions can be listed as follows:

- A complete systematic survey is provided for MIMO VLC-based studies available in the literature. We first describe the VLC working principle, different techniques of MIMO, and the channel model.
- We describe the existing works by grouping related works into a category and describing working methods and results.
- Machine learning approaches are also described for MIMO VLC approaches, and future directions are provided.

The rest of the paper is organized as follows. Section 2 describes the basics of the VLC technique, channel model, and MIMO communication model. Section 3 describes the different techniques available in MIMO communication. Section 4 describes the different studies conducted in the previous literature regarding MIMO VLC by problem category. Machine learning approaches that have been deployed in MIMO VLC are described in Section 5. Future directions are described in Section 6, and conclusions are given in Section 7.

## 2. MIMO Communication Theory for VLC

MIMO communication has been extensively studied in RF communication as compared to VLC. However, in the past 10 years, MIMO VLC has also been studied in a comprehensive way. Table 2 shows the gradual improvement of data rate using MIMO VLC.

### 2.1. VLC Working

**Figure 2:**
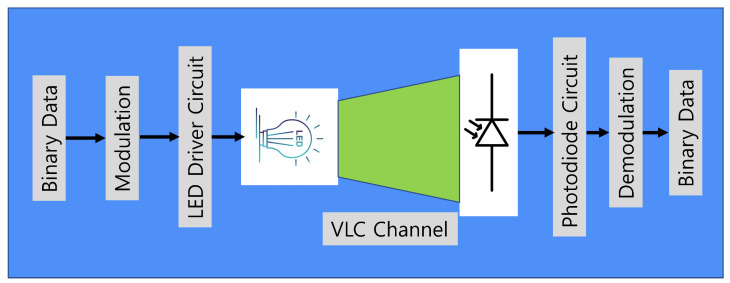
Visible light communication system and elements.

**Figure 3:**
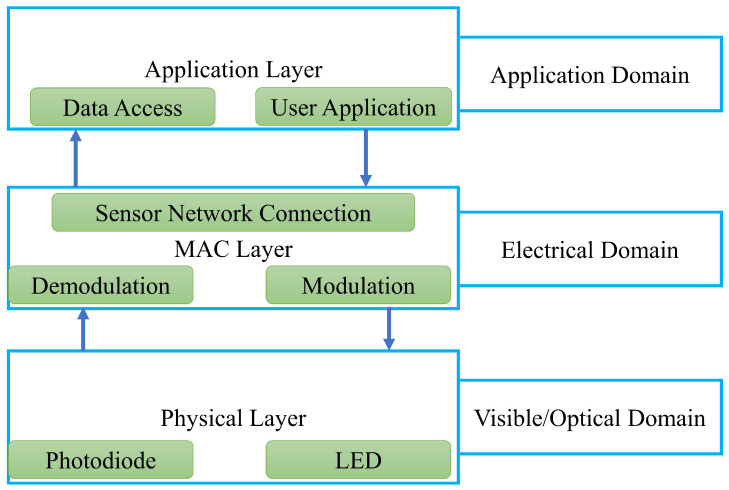
Visible light communication framework.

VLC-based communication is a promising solution for next-generation wireless connectivity with data security [67]. The communication system and elements for a typical VLC are shown in Figure 2. In the beginning, binary data which are needed to be transmitted were prepared from data sources or sensors [68]. Next, any of the modulation techniques can be chosen for communication. The common modulation technique includes on-off keying (OOK), pulse position modulation (PPM), multiple pulse position modulation (MPPM), pulse amplitude modulation (PAM), and pulse width modulation [6]. Other complex modulations are also available for VLC, and readers are encouraged to read the referenced paper for more details [69]. Next, the modulated signals are transmitted through an LED transmitter. All the transmitted signals are positive and real in nature, as LEDs can not transmit imaginary values. The LEDs now work as a data transmitting device that simultaneously illuminates and transmits. After passing through the VLC channel, the light signals are received by photodiodes which are typically placed directly toward the LEDs as shown in Figure 2. Next, the receiver circuit amplifies the signal, and then it is transmitted to the receiving microprocessor unit (MCU). At this stage, demodulation is performed, and the original bits are reproduced at the receiver end. The transmitter and receiver should use the same frequency for modulation and demodulation. As the distance between the transmitter and receiver increases, the error in the channel increases due to a reduction in illumination [70]. The VLC communication framework is shown in Figure 3. The physical layer contains photodiodes and LEDs. In this layer, data is converted into the optical domain and again converted into the electrical domain. The upper layer is the MAC layer, which controls the communication of the channel. Modulation and demodulation are performed in this layer, and also network control commands are given from this layer. The application layer is the access layer for users. Here the transmitted or received data can be accessed from the outside world.

### 2.2. VLC Channel Model

LED illumination is the key factor in VLC-based communication. The luminous intensity can be expressed as follows [15]:

(1) $$i=\frac{d\phi }{d\omega },$$

where ϕ is the spatial angle and ω is the luminous flux. The luminous intensity for an angle δ can be defined as follows [71]:

(2) $$i\left(\delta \right)=i\left(0\right)\cos^{m}\left(\delta \right),$$

where i(0) is the center illuminance and *m* is the Lambertian emission. The horizontal illumination can be expressed as follows [71]:

(3) $$e_{hor}=\frac{i\left(0\right)\cos^{m}\left(\delta \right)}{d^{2}\cos\psi },$$

where δ is the transmitted signal angle or irradiance angle, ψ is the receiver angle or incidence angle and *d* is the distance between LED and receiver photodiode. The Lambertian emission can be defined as follows:

(4) $$m=\frac{-\ln2}{\ln(\cos(0.5\alpha ))},$$

where α is the LED illumination angle at half power. The optical power calculation of the received data is crucial. The received DC gain can be expressed at the photodiode as follows:

(5) $$h=\left\{\begin{array}{cc} \frac{(m+1)A}{2\pi d^{2}}\cos^{m}\left(\delta \right)T\left(\psi \right)g\left(\psi \right)\cos\left(\psi \right), & 0<\psi <\phi_{c}\\ 0, & \psi >\psi_{c}, \end{array}\right.$$

where *A* is the area of the photodiode, $\psi_{c}$ is the field of view of the receiver, *d* is the distance between LED and PD, T(ψ) is the gain of the optical filter, and g(ϕ) is the gain of the optical concentrator. The optical concentrator gain can be expressed as follows:

(6) $$g\left(\psi \right)=\left\{\begin{array}{cc} \frac{n^{2}}{\sin\psi_{c}}, & 0\leq \psi \leq \psi_{c}\\ 0, & 0\geq \psi_{c} \end{array}\right.$$

where *n* is the refractive index. The received optical power $p_{r}$ can be obtained as follows:

(7) $$p_{r}=H.p_{t},$$

where $p_{t}$ is the transmitted power.

### 2.3. MIMO VLC

**Figure 4:**
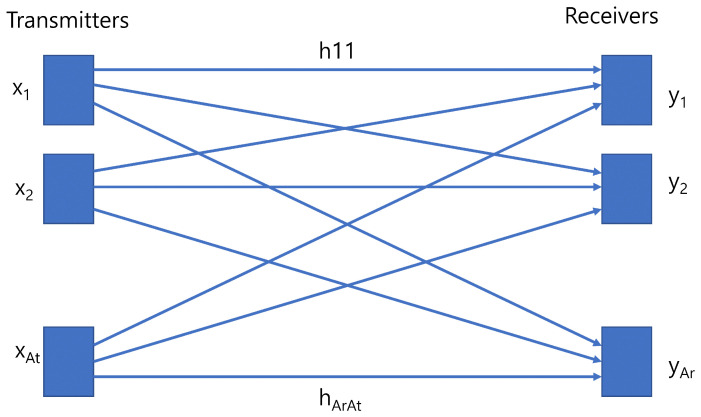
MIMO Communication Channel.

A narrowband MIMO point-to-point channel model as shown in Figure 4 with $A_{t}$ transmitters and $A_{r}$ receivers can be expressed as follows [72]:

(8) $$\left[\begin{array}{c} y_{1}\\ y_{2}\\ \vdots \\ y_{A_{r}} \end{array}\right]=\left[\begin{array}{ccc} h_{11} & \cdots & h_{1A_{t}}\\ \vdots & \ddots & \vdots \\ h_{A_{r}1} & \cdots & h_{A_{r}A_{t}} \end{array}\right]\left[\begin{array}{c} x_{1}\\ x_{2}\\ \vdots \\ x_{A_{t}} \end{array}\right]+\left[\begin{array}{c} n_{1}\\ n_{2}\\ \vdots \\ n_{A_{r}} \end{array}\right],$$

where $y=y_{1},\cdots ,y_{A_{r}}$ is the $A_{r}$ number of receiver, $x=x_{1},\cdots ,x_{A_{t}}$ is the $A_{t}$ number of transmitter, $H=A_{r}\times A_{t}$ is matrix of channel gain and $n=n_{1},\cdots n_{A_{r}}$ is the noise vector. Thus, (8) can be written as follows:

(9) y=Hx+n,

where y is the received signal, H is the channel matrix, x is the transmitted signal and n is the noise occurring in communication channel usually considered as additive white Gaussian noise. The sum of the ambient light and thermal noise is *n*, which is considered as zero mean and variance as follows:

(10) $$\sigma^{2}=\sigma_{shot}^{2}+\sigma_{thermal}^{2},$$

where, $\sigma_{shot}$ is the shot noise and $\sigma_{thermal}$ is the thermal noise occurring in VLC channel.

Apart from conventional RF communication, VLC data transmission is different as it depends on intensity modulation direct detection. VLC has two different receiver architectures of imaging and non-imaging for receiving MIMO signals [73]. In imaging receiver architecture, an array of photodiodes are employed to capture the incoming signal. The imaging receiver has advantages that all photodiodes share a common concentrator which makes the receiver size small, and all photodiodes are laid in a single array which increases the receiver elements [74]. It can increase the optical gain for communication. On the other hand, non-imaging receivers are made of individual circuit components that precise alignment is not required [75]. The channel matrix element for the MIMO VLC setup can be expressed as follows:

(11) $$h_{ij}=\left\{\begin{array}{cc} \sum_{k=1}^{K}\frac{\left(m+1\right)A^{j}}{2\pi d_{ijk}^{2}}\cos^{m}\left(\delta \right)\cos\left(\psi_{ij}\right) & 0\leq \psi_{ij}\leq \psi_{c}\\ 0 & 0\geq \psi_{c}, \end{array}\right.$$

where $A^{j}$ is the area of the *j*th receiver, $d_{ijk}$ is the distance between the *k*th LED of the *i*th transmitter and *j*th receiver. Thus, the channel matrix can be formed as follows:

(12) $$H=\left[\begin{array}{cccc} h_{11} & h_{1j} & \cdots & h_{1A_{t}}\\ h_{i1} & h_{ij} & \cdots & h_{2A_{t}}\\ \cdots & \cdots & \cdots & \cdots \\ h_{A_{r}1} & h_{i2} & \cdots & h_{A_{r},A_{t}} \end{array}\right]$$

## 3. MIMO Communication Types

Several MIMO communication techniques are developed for data transmission. In this section, we describe each technique in detail and the works associated with each technique.

### 3.1. Repetition Coding

**Figure 5:**
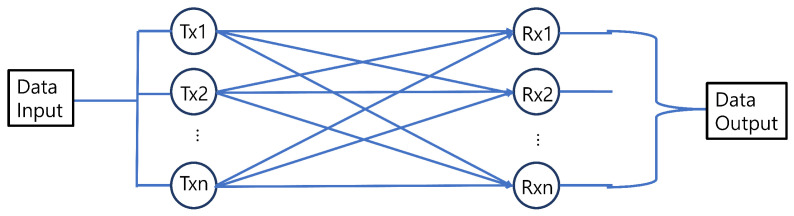
RC MIMO that each of the transmitters sends same data signal.

In repetition coding (RC), the same data stream or signal is transmitted through multiple antennas [76]. RC is the simplest form of MIMO communication and achieves good performance in free space optical communication because of transmit diversity. RC mechanism is demonstrated in Figure 5, where each transmitter sends the same data signal.

The authors in [77] showed that RC could perform better than orthogonal space-time block codes (OSTBCs) like the Alamuouti scheme and single-input-multiple-output (SIMO) configurations. RC was investigated in [78] with angular diversity receiver (ADR) based MIMO VLC for imperfect channel state information (CSI). The results showed that ADR-based MIMO VLC has better error performance as compared to MISO VLC. Adaptive bit and power loading for OFDM VLC MIMO system was proposed in [79]. An adaptive algorithm was proposed to enhance spectral efficiency by selecting modulation order, power level, and MIMO antenna mode. The authors in [80] studied the effect of RC in VLC in a 5 m × 5 m × 3 m room with 4 transmitters. Simulation studies represented that RC can only have better bit error rate (BER) performance with low spectral efficiency requirements as compared to SMP. The theoretical BER for RC can be obtained from (13) [76], where *L* is the modulation level of PAM, *Q* is the Q-function, E is the emitted electrical energy, $n_{0}$ is the noise power spectral density, $N_{t}$ is the number of transmitters, $N_{r}$ is the number of receivers, and $h_{n_{r}n_{t}}$ is the channel gain of a transmitter–receiver pair.

(13) $$\begin{array}{c} RC_{BER}\geq \frac{2(L-1)}{L\log_{2}\left(L\right)}Q\left(\frac{1}{L-1}\sqrt{\frac{E}{n_{0}N_{t}}\sum_{n_{r}=1}^{N_{r}}{\left(\sum_{n_{t}=1}^{N_{t}}h_{n_{r}n_{t}}\right)}^{2}}\right) \end{array}$$

(14) $$\begin{array}{c} SM_{BER}\leq \frac{1}{LN_{t}\log_{2}\left(LN_{t}\right)}\sum_{l^{\left(1\right)}=1}^{L}\sum_{n_{t}^{\left(1\right)}=1}^{N_{t}}\sum_{l^{\left(2\right)}=1}^{L}\sum_{n_{r}^{\left(1\right)}=1}^{N_{r}}d_{H}\left(b_{l^{\left(1\right)}n_{t}^{\left(1\right)}},b_{l^{\left(2\right)}n_{t}^{\left(2\right)}}\right).\\ Q\left(\sqrt{\frac{r^{2}T_{s}}{4\;n_{0}}}\sum_{n_{r}=1}^{N_{r}}{\left|I_{l(2)}^{SM}h_{n_{r}n_{t}^{\left(2\right)}}-I_{l(1)}^{SM}h_{n_{r}n_{t}^{\left(1\right)}}\right|}^{2}\right) \end{array}$$

### 3.2. Spatial Modulation

**Figure 6:**
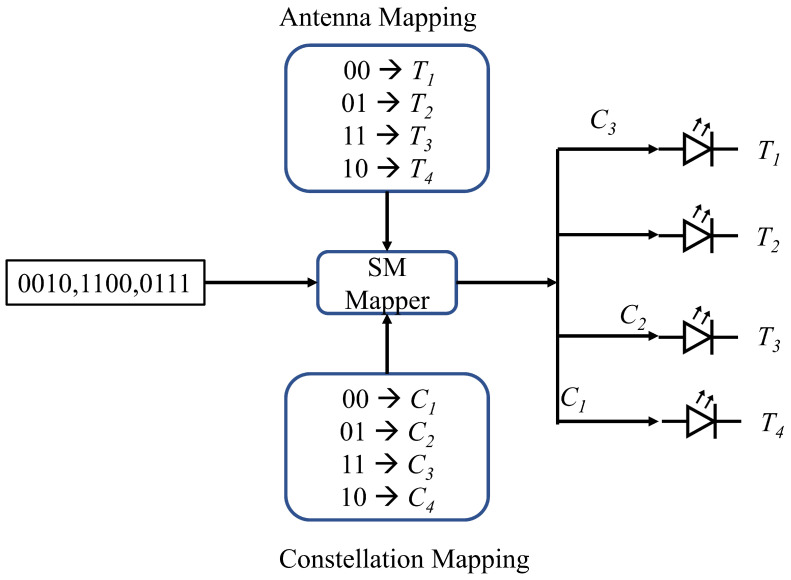
SM example for MIMO communication for each of the transmitters.

Spatial Modulation (SM) is a combined technique of both MIMO and digital modulation proposed in [81]. SM works by mapping the information bits in two steps [82]. First, the information bits are mapped into a constellation point. Second, an antenna is chosen for transmitting a particular bit pattern from a set of antennas. An example of SM is shown in Figure 6. Four constellation points and four antennas are shown for data transmission. Three input bit patterns are transmitted through the channel, and each transmission constellation and antenna are selected on the right side after the SM mapper. Here $\left\{T_{1},T_{2},T_{3},T_{4}\right\}$ represents four antenna indices and $\left\{C_{1},C_{2},C_{3},C_{4}\right\}$ represents four constellation points. Optical SM was studied in [83] that multiple transmitters are spatially separated in a room environment, and at one instance, only one transmitter is activated. Depending upon the input bit sequence, the transmitter is selected. The performance of the optical SM is compared with OOK, 4-PPM and 4-PAM modulations, and then simulation results show that optical SM has a similar BER performance to OOK. The BER of SM can be calculated from (14), where *L* is the number of levels in PAM modulation, $N_{t}$ is the number of transmitters, $N_{r}$ is the number of receivers, $b_{l^{\left(1\right)}n_{t}^{\left(1\right)}}$ is the bit assignment when the transmitter intensity is $I_{l(1)}^{SM}$, $b_{l^{\left(2\right)}n_{t}^{\left(2\right)}}$ is the bit assignment when the transmitter intensity is $I_{l(2)}^{SM}$, $d_{H}$ is the Hamming distance between the two parameters, *r* is the optical to electrical conversion coefficient, $T_{s}$ is the symbol duration, and $h_{n_{r}n_{t}^{\left(1/2\right)}}$ is the channel gain.

#### 3.2.1. Adaptive Spatial Modulation

One of the limitations of SM is the transmitter diversity gain, and to combat this issue, adaptive spatial modulation (ASM) is proposed for BER improvement. The authors in [84] proposed ASM for achieving better performance under a fixed data rate. The main idea is that the modulation orders are assigned to the transmit antennas selected by the switching unit. In a slowly varying channel, the adaptive unit in the receiver computes the optimum candidate for transmission and sends this information to the transmitter through a low-bandwidth feedback path. Based on the feedback information, the transmitter’s corresponding modulation order for the next data transmission is determined. Different forms of adaptive generalized spatial modulation (GSM) have been studied in previous studies. Chromaticity-adaptive (CA) GSM method for MIMO VLC was proposed in [85]. The optimal QLED combination is selected by CA-GSM based on a multi-color constellation designed by Taylor approximation. Another approach called channel-adaptive bit mapping (CABM) was proposed in [86].

#### 3.2.2. Generalized Spatial Modulation

In GSM, more than one transmit antenna sends the same complex symbol [87]. Information is transmitted by activating a combination of antennas and symbols from the signal constellation. It increases the spectral efficiency as compared to SM. At each transmission, the number of possible active antennas is Nc=NtNu, where $N_{t}$ is the total number of antennas and $N_{u}$ is the number of active antennas for transmitting data. To fit the binary data, the number of transmitter antenna combinations should be at the power of 2. Thus, $N_{c}=2^{c_{a}}$, where ca=log2NtNu and $\left\lfloor .\right\rfloor$ is the floor operation. Thus, $C_{a}$ bits can be mapped to the antenna combinations and let us consider $c_{t}$ bits are modulated by using *M*-quadrature amplitude modulation (QAM), and then total bits can be transmitted:

(15) cb=ca+ct=log2NtNu+log2M.

The performance of GSM in indoor VLC scenario was investigated in [88]. Four different MIMO schemes are considered as SMP, SM, space shift keying (SSK), and generalized space shift keying (GSSK). An analytical upper bound on BER for GSM with maximum likelihood detection is derived. The simulated BER shows that GSM can achieve favorable performance as compared to other MIMO schemes. Another study focused on power efficiency using collaborative constellation (CC) GSM proposed was proposed in [89]. The key idea is to find a set of constellations with active space CC with minimum power as an optimization problem. The simulation results show average pairwise error probability is less for the proposed scheme as compared to conventional GSM. A support vector machine (SVM) based GSM detector was proposed in [90]. The optimization problem of quadratic convex programming is solved by training the parameters of SVM and providing comprehensive results.

(16) $$\begin{array}{c} SMP_{BER}\leq \frac{1}{L^{N_{t}}\log_{2}\left(L^{N_{t}}\right)}\sum_{m^{\left(1\right)}=1}^{L^{N_{t}}}\sum_{m^{\left(1\right)}=1}^{L^{N_{r}}}d_{H}\left(b_{m^{\left(1\right)}},b_{m^{\left(2\right)}}\right).Q\left(\sqrt{\frac{r^{2}T_{s}}{4\;n_{0}}}||H\left(s_{m^{\left(1\right)}}-s_{m^{\left(2\right)}}\right){\left|\right|}_{F}^{2}\right) \end{array}$$

### 3.3. Spatial Multiplexing

**Figure 7:**
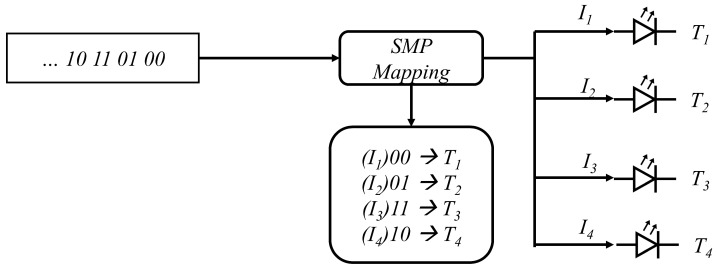
SMP example for MIMO communication four antenna configurations .

In spatial multiplexing (SMP), each of the transmitting antenna LEDs transmits different data streams (i.e., independent of others) simultaneously [91]. Thus, SMP has higher spectral efficiency as compared to RC and SM [92]. Figure 7 shows the SMP technique with four antenna configurations. From the left side, the data stream is inserted into the SMP mapping system, and two bits are selected for transmitting through one antenna. So, for $2^{n}$, antennas can transmit *n* bits at one time. The spectral efficiency of SMP is $N\log_{2}\left(M\right)$ bits/s/Hz, where *N* is the number of transmitting antennas. In SMP, multiple data streams are transmitted, so there is a high probability of multi-channel interference, which can cause performance degradation. In [76], the authors showed that SMP could provide superior performance enhancement for SMP configurations in optical communication. In addition, imaging receivers can achieve better performance gain for SMP [93]. The BER expression for SMP is shown in (16), where *L* is the number of levels in PAM modulation, $N_{t}$ is the number of transmitters, $N_{r}$ is the number of receivers, $b_{m^{\left(1\right)}}$ is the bit assignment from signal vector $s_{m^{\left(1\right)}}$, $b_{m^{\left(2\right)}}$ is the bit assignment from signal vector $s_{m^{\left(2\right)}}$, $d_{H}$ is the Hamming distance between the two parameters, *r* is the optical to electrical conversion coefficient, $T_{s}$ is the symbol duration, and H is the given knowledge of the channel matrix. The study in [92] presented a comparison in terms of BER between SMP and optical spatial modulation (OSM) in indoor environments. The BER results illustrate that SMP outperforms OSM in terms of both the size of the region in which a receiver can achieve low BER and the BER at typical receiver positions. The authors in [94] proposed a superimposed odd-order 32QAM constellation scheme in 2 × 2 MIMO VLC systems to achieve multiplexing gain in highly correlated channels. Two independent signals from 4QAM and 8QAM are superposed to make a 32QAM constellation signal combined with SMP. An SMP point-to-point VLC was considered in [95], with M-level PAM and SMP. By utilizing an SVD-based low complexity scheme, analytical expression was derived for power and bit allocation subject to maximizing the lower bound capacity. Another 64QAM constellation scheme in [96] was proposed for 2×2 MIMO configuration for SMP. The experimental results show that the proposed scheme achieves better BER performance than the traditional superposed 64QAM constellation schemes. The SMP can give additional bandwidth as compared to RC and SM, and, thus, most research studies have focused on performance improvement.

## 4. MIMO Communication Study Categories

In this section, we categorize the VLC MIMO studies based on different problems and provide a brief description of each category.

### 4.1. Precoder Design

Precoder design is a technique to reduce interference among co-channels through spatial processing by improving spectral efficiency. In [97], the authors proposed a linear precoder matrix design in the transmitter and linear equalizer at the receiver to reduce mean-square error in transmitted data and received data. Simulation results show the effectiveness of the proposed technique for known CSI and unknown CSI. Moreover, the proposed system can combat the uncertainties case by the channel estimation imperfection. A joint precoder and equalizer design were proposed in [98] for multi-user multi-cell MIMO communication. An optimization approach was formulated by minimizing mean-squared error (MSE) under unique optical power constraints when real-valued and non-negative signals are transmitted. The authors in [99] proposed decision feedback equalization based on point-to-point MIMO communication. Geometric mean decomposition, which decomposes multiple parallel channels with equal gain and uniform decomposition, improves the capacity by incorporating optimized power allocation. Block bi-diagonalization (BBD) enabled communication was proposed in [100] for mitigating interference in MIMO VLC. The proposed BBD scheme can mitigate different noises, including thermal, shot, and phase noise. QAM modulation was used to transmit data and BER results were presented for three different scenarios that have different dimensions and SNR ranges.

### 4.2. Channel Estimation

The performance of the wireless channel largely depends on the channel estimation. In the case of MIMO communication, the transmitting and receiving antennas are multiple. Thus, accurate channel estimation is necessary for superior performance. Different methods have been proposed in the literature for estimating the VLC MIMO channel. The most common techniques for estimating channels are the least square (LS) and minimum-mean-squared-error (MMSE) [101,102]. Compressive sensing-based channel estimation was proposed in [103]. Due to the sparse characteristics in the VLC channel, compressed sensing-based channel estimation was considered for 2×2 MIMO-OFDM. The experiment results show that the proposed channel estimation can improve BER with reduced pilot tones. Another study in [23] proposed a channel estimation scheme for mitigating interference for the angular receivers. In the first stage, pilot symbols are transmitted to determine the transmitter’s identity. Next LS scheme is used for channel estimation and maximum likelihood is used for detection in the receiver. Optimal code with short length for estimation of MIMO VLC channel was proposed in [104]. A recursive algorithm is used to generate optimal pilot matrices depending on the number of LEDs. In [105], authors used a generalized LED (GLIM-OFDM) VLC system for channel estimation.

### 4.3. Multi-User Massive MIMO

Multi-user communication is desirable for serving many users at the same time. The greater challenge is to separate the received data bit streams for different users. The study in [106] proposed a block diagonalization precoding algorithm for minimizing multi-user interference. BER performance of user mobility was investigated by using the proposed method with a 100 Mbps data rate. Multi-user communication by employing OFDM-based VLC communication was proposed in [107]. For every OFDM subcarrier, the precoding matrix is calculated in the frequency domain to eliminate multi-user interference. The authors in [108] used different pilot arrangements in spatial, frequency, and time domains to obtain a global channel matrix taking advantage of the indoor environment geometry and layout of luminaries. OFDM was employed to determine the maximum uplink and downlink data rate of the proposed system to support muli-user communication. Hybrid three-dimensional multiple access (3DMA), including frequency, space, and power, was proposed in [109], for multi-user MIMO VLC. To leverage 3-dimensional multiple access, the first different user group is created, and each user group is divided into multiple user pairs. The sum rate maximization was derived by power-domain superposition coding and the corresponding optimal power allocation strategy for each user pair. In [61], single-user and multi-user VLC was studied in an indoor environment. For the demodulation of data in single-user, maximum ratio combining (MRC) was used, and for multi-user spatial multiplexing, MRC and transmitter/receiver diversity were used. The data rate achieved for a single user is 4 Gbps, and for a multi-user, 1.5 Gbps. Again in [110], authors proposed optical OFDM photodiode selection assisted multi-user MIMO communication which can reduce VLC channel correlations between different photodiode receivers and, thus, provide a reliable link. The simulation results show that the proposed system can achieve good BER in low SNR values. DenseVLC was proposed in [111] for a cell-free approach by employing densely distributed LEDs in the service area. A power budget optimization problem was also formulated to efficiently control and design the transmitter and receiver (i.e., hardware design). Three experimental scenarios were presented interference-free and no-dominating transmitter communication with interference and no dominating transmitter, and finally with interference and dominating transmitter. The experimental results show good performance of the proposed system in three different scenarios. The authors in [112] studied the secrecy performance of multi-user MIMO VLC with broadcast channels using confidential messages. The transmitting user message is sent by considering only one valid user, and other users are eavesdroppers. Different secrecy performance measures were investigated, including the max-min fairness, the harmonic mean, the proportional fairness, and the weighted fairness (WF). The proposed system can achieve a good performance in comparison to the zero forcing algorithm.

### 4.4. Angle Diversity of Receiver

Multiple receivers can be placed at different angles to increase the overall gain in the MIMO VLC system. The authors in [113] proposed a receiver structure utilizing angular and spatial diversity to achieve full mobility and protection from signal blocking. The recipient has an array of photodiodes with transimpedance amplifiers connected to a decision device that generates binary address depending upon the received signal strength indicator (RSSI) signal. The multiplexer connected to the decision device generates the original bits upon receiving the address of the highest RSSI signal. Nuwanpriya et al. [114] proposed diversity receivers for MIMO named pyramid receiver and hemispheric receiver to achieve high-rank MIMO channel. Simulation results show that both receivers have good performance in channel capacity and BER. A mobile receiver has angular diversity detectors for the MIMO channel considered in [115]. Channel throughput was improved by considering RC, SM, and SMP in a small room scenario. The proposed detector can provide capacity improvement as compared to vertically oriented receivers. Another study in [23] proposed an adaptive diversity receiver with the least square channel estimation with a maximum-likelihood equalizer for performance enhancement. Pyramid shape receiver was considered for receiving signals from different directions and distances.

### 4.5. NOMA-Based MIMO

Non-orthogonal multiple access (NOMA) is an efficient technique for serving multiple users [116]. NOMA enables multiple users to share time and frequency resources in the same spatial layer via power domain or code domain [117]. VLC-based communication has also adopted the NOMA strategy for enhancing performance. Power domain (PD) NOMA has the advantages like user fairness, improved spectral efficiency, low transmission latency, and higher cell-edge throughput. The study in [118] experimentally demonstrated NOMA-based MIMO communication with single carrier transmission and frequency domain successive interference cancellation. In [119], the authors proposed offset quadrature amplitude modulation (OQAM)-OFDM based MIMO-NOMA for multi-user VLC, and the data rate of 3.2 Gbps was achieved. To reduce the computational complexity, the study in [120] proposed normalized logarithmic gain ratio power allocation (NL-GRPA), which is effective for more than five users in the service area. Simulation results verify the effectiveness of the proposed scheme in terms of achievable sum rate as compared to GRPA. Again in [121], normalized gain difference power allocation was proposed for efficient and low complexity power allocation in the MIMO-NOMA-VLC system. The sum rate performance for the 2 × 2 system was evaluated via a simulation study. Another study conducted in [122] evaluated sum rate gain for LOS and LOS+NLOS in a single reflection environment. Numerical results show that NOMA with NGDPA attains a 16.71% refined sum rate than NOMA with GRPA in the LOS environment and 18.22% in the combined LOS and NLOS single reflection environment at the edge of the room when the standardized offset is 1. In [123], authors analyzed the total capacity of 2 × 2 MIMO VLC system using GRPA and NGDPA algorithms. The performance comparison was taken by system coverage, user location, and the number of users. For increasing coverage, the capacity of NGDPA outperforms GRPA; for less than 1.2 m distance, GRPA performs well as compared to NGPDA, and for increasing the number of users, NGPDA is better than GRPA. The authors in [124] used zero forcing equalizer with successive interference cancellation (ZF-SIC) and minimum mean square error equalizer with successive interference cancellation (MMSE-SIC) to improve the BER performance of the NOMA MIMO system. It is concluded that MMSE-SIC improved the BER by 3 dB as compared to ZF-SIC. A multi-user NOMA transmission scheme was proposed in [125], where the users having high correlation among their channel gain vector are grouped into a single cluster. The simulation result shows that the proposed method can provide better performance as compared to ZF and BD in terms of spectral efficiency. L-PPM modulated NOMA-VLC was proposed in [126], for determining the error probability of two-user and three-user scenarios. L-PPM modulation can outperform OOK modulation and can offer optimal performance at a power allocation coefficient of 0.3.

### 4.6. Optical Camera Communication Using MIMO

**Figure 8:**
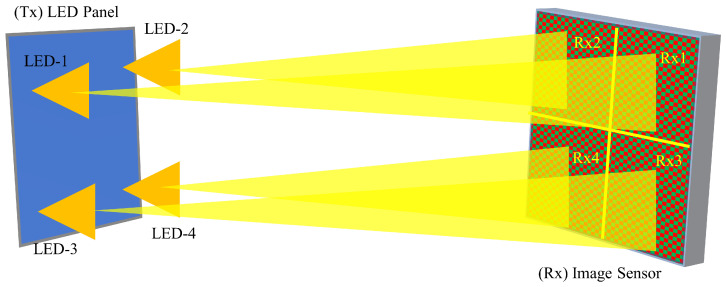
4×4 MIMO optical camera communication.

Optical camera communication (OCC) is also an associated technology of VLC where the receiver is used as a camera device [127,128]. A MIMO optical camera communication scenario is shown in Figure 8, where the receiver is an array of photodiodes. As the number of smartphones has increased dramatically, this technology can provide additional advantages to users. The authors in [129] proposed a MIMO communication system with RGB-LEDs as transmitters and a single camera as a receiver. Two different colors of red and blue are used for data and anchor transmission. Hadamard matrix was chosen in LED detection for recovering the bit from image processing. The authors in [130] presented a VLC MIMO study by OCC using an adaptive target detection algorithm at the receiver end. The number of LEDs in the transmitter is considered 8×8, and the number of photodiodes in the receiver is 8. Each of the links in the transmitter and receiver transmits different data streams in parallel. The transmission distance can be achieved from 6 m to 14 m. Han et al. [131] proposed fixed-scale pixelated MIMO VLC system. The data are transmitted by space-to-angle mapping, and data are transmitted in the angular domain rather than space. This can achieve constant focus on the receiver, and, thus, re-focusing is not necessary. The study in [132] used an array of 8 × 8 LEDs as a transmitter and a Raspberry Pi camera module as a receiver. To transmit data, 64 LEDs are used as camera pixels, and each represents one data bit. The receiver camera processes the image and recovers the bits, and the modulation technique used in the experiment is OOK. However, as the distance increases, the number of captured bits is reduced.

### 4.7. Constellation Design

Constellation is a representation of signal after modulation by any digital modulation technique. To enhance the bit transfer rate, the constellation can be designed in a new way. A collaborative constellation design was presented in [133]. Unipolar r-levels pulse amplitude modulation (r-PAM) symbols of the transmitters are designed for data transmission. The constellation design achieves optimal power-efficient subject to a fixed minimum Euclidean distance jointly. Guo et al. in [94] proposed a superposed odd-order 32QAM constellation scheme for 2 × 2 MIMO VLC. Two transmitters transmit 4QAM and 8QAM signals to combine a 32QAM signal in the receiver. Three types of geometric representations were chosen for 8QAM, square-shaped, rectangular-shaped, and circle shaped. The experimental study shows that under different peak-to-peak voltage conditions, the BER rate can be improved as compared to traditional constellations. Another study in [96] extended the previous study to 64QAM by employing 8QAM signals, and each constellation is shifted by 90°. Forward error correction BER threshold 3.8 $\times 10^{-3}$ was achieved with a peak-to-peak LED voltage improvement 0.06 to 4 V. $2^{n}$ order 4QAM signal is proposed in [134] with a maximum rate of 3 Gbps.

### 4.8. Underwater Communication

**Figure 9:**
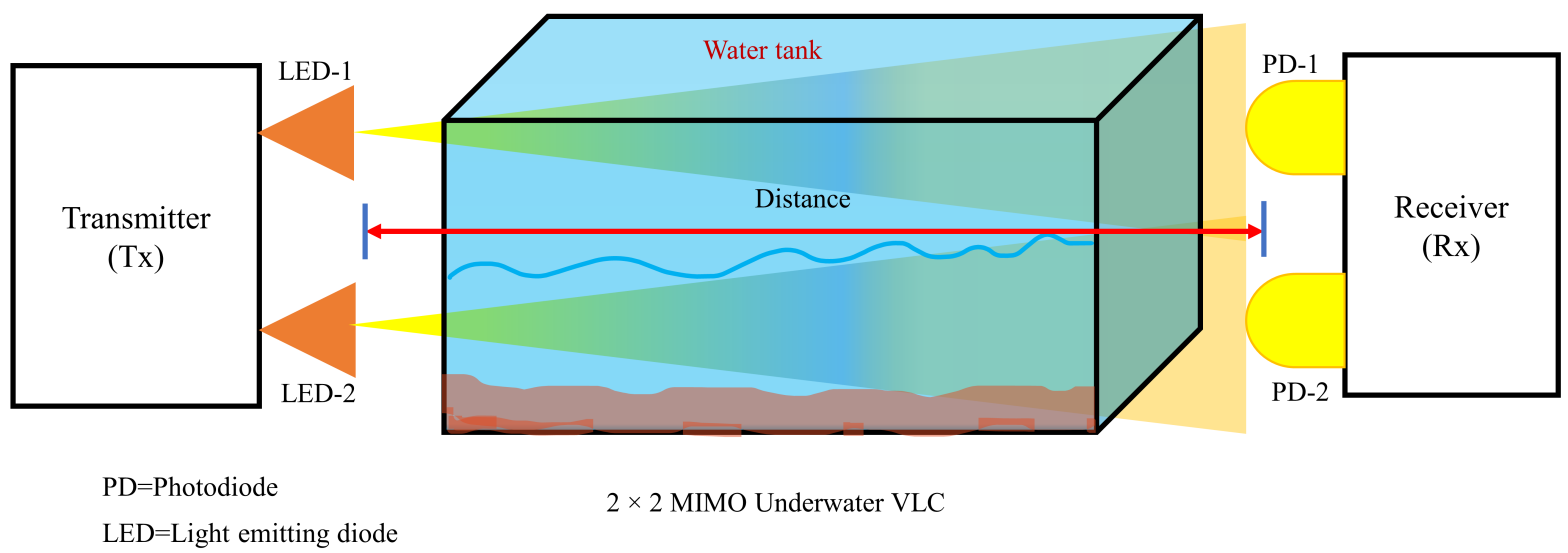
2×2 underwater MIMO VLC communication.

Underwater communication has also attracted significant research attention among researchers for many potential applications. Optical communication has been considered as a potential candidate for implementing communication in underwater environments due to high-speed communication [135]. The underwater communication experiment setup with 2 × 2 settings is shown in Figure 9. An experimental study was performed in [136] using MIMO-OFDM for underwater communication. 2 × 2 MIMO configuration was used for achieving a 2 m distance with a 33.691 Mbps data rate. In addition, turbid water was used to compare RC OFDM, Alamouti-OFDM, and MISO-OFDM, and among these techniques, Alamouti-OFDM is more resistant. In [137], the authors presented work on MIMO link over a vertical turbulence channel model. The outage probability of the MIMO VLC link over cascaded log-normal channels and diversity gain was derived in terms of the number of transmitters and receivers. The performance was considered for different transmitter/receiver apertures for plane and spherical waves, and the number of transmitter/receiver pairs can increase the performance in seawater. The authors in [138] presented an imaging MIMO system for underwater communication to combat absorption and scattering by using spatial correlation. Simulation results show a 12 dB gain as compared to non-imaging MIMO in BER performance. The study in [139] experimented with different modulation schemes in MIMO communication in coastal water. A comprehensive study was performed by Jamali et al. [140] for VLC MIMO communication considering channel degrading effects, including absorption, scattering, and turbulence-included fading. The authors in [141] analyzed the BER performance of Log-normal, gamma, and Weibull distribution channels for underwater MIMO communication.

### 4.9. Vehicle-to-Vehicle Communication

**Figure 10:**
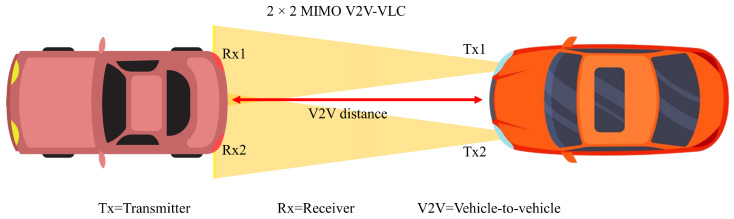
2×2 vehicle-to-vehicle MIMO VLC communication.

To establish wireless communication between vehicles, VLC can be a promising candidate as light sources are already embedded in vehicles. The high presence of LEDs in outdoor and on-vehicle environments makes the use of VLC a natural opportunity for V2V and V2I communications for ITS systems [142]. A typical example of vehicle-to-vehicle is shown in Figure 10, where the two headlights are used as transmitters, and the receivers are situated in the brake lights. The opposite can also be possible when the brake lights can transmit data, and the receiver can be situated in the front headlight. The study in [143] reported point-to-point and decode-and-forward relaying-based cooperative VLC. RC and SM with DC-biased optical (DCO)-OFDM different modulation orders were investigated, including 8QAM, 16QAM, 64QAM, and 256QAM. RC-based direct communication outperforms SM-based communication, and in higher modulation order, SM outperforms RC in longer distance communication. The study in [144] proposed MIMO VLC-based vehicular communication using frequency diversity. In the receiver, a filtering process is employed for receiving specific frequency range data, and other data elements are canceled out. It helps to receive specific LED frequency data for demodulation. Li et al. in [65] extensively experimented with vehicle-to-vehicle communication in long-distance and high data rate applications. Different scenarios were considered, and corresponding BER was reported in the study. Bit loading algorithm and nonlinear equalization were utilized to improve the performance of the proposed system. Another study in [145] conducted different MIMO configurations 2 × 2, 2 × 3, and 2 × 4 channel modeling study. Increasing the receiver number can reduce the amount of BER for V2V scenarios.

### 4.10. Other MIMO VLC Studies

Different studies are also conducted which do not fall in the above-mentioned category. To increase the field of view (FOV) and diversity gain, a hemispherical lens was proposed in [146]. Simulation results were obtained for the receiver, and for a typical indoor scenario, FOV increased as large as 70 degrees for the angle of the incident. MIMO VLC multipath reflection effect was studied in [147]. Space division multiple access (SDMA) for indoor spatial multiplexing-based MIMO-VLC was proposed by Chen et al. [148]. SDMA can support multiple users in an indoor single-cell multiuser MIMO VLC by dividing them into user groups. The study in [149] examined MIMO communication in an industrial environment. A manufacturing cell of the production facility was used for the experiment with 8 × 6 MIMO configuration. The experiment result shows that the channel significantly varies in the spatial domain with abrupt changes in SNR ranging from 10–20 dB. Another industrial experiment environment was considered in [150] that developed a MAC protocol based on space division multiple access. The system was designed to have one central transmitting unit, which is connected to other optical-fronted devices covering a large area. A pixelated transmission system was proposed in [131], which transmits time-varying image code to the receiver. The receiver is used as a commercial camera to receive and decode the transmitted data along with the location information of the transmitter. The experiment was conducted using an LCD display and a high-speed CMOS camera, and data can be transmitted at a 1 m distance. Singular value decomposition (SVD) for MIMO VLC was studied in [151]. This proposed system maximizes the data rate while maintaining the target illumination and allowing the channel matrix to vary to support indoor VLC deployment mobility. Another study in [47] proposed non-imaging 2 × 2 MIMO Nyquist single carrier for indoor VLC. For demultiplexing and post-equalization simultaneously of the received signal, a frequency domain equalization method was proposed. In [152], the authors implemented camera on-off keying for MIMO communication. Two LEDs transmit data and the demodulation was performed by determining the region of interest by image processing. An effective receiver design was proposed in [153]. An optimization method was proposed to maximize the minimum Euclidean distance of the received signal. A compressive sensing channel estimation approach was proposed in [105]. Higher-order statistics correntropy was suggested for generalized LED index modulation of the OFDM system. An efficient modulation technique for MIMO named extended spatial index LED was proposed in [154]. Variable power allocation is used by different transmitter LEDs to transmit information to the receiver. To detect the signal, a maximum a posteriori estimator was proposed to cope with the variation in the channel paths (LEDs) number and the real part power allocation. To increase the illumination distribution and improve BER for indoor VLC, a scheme called LEDs inclined MIMO (LIM) was proposed in [155]. Integer-forcing (IF) lattice decoding-based transceiver design was proposed in [156]. The design considered the creation of an integer matrix that can be invertible over a one-dimensional lattice; next, a method was used to obtain a new integer matrix that performs better than the previous one. In addition, transmit and receive matrices were designed by the gradient method or projected gradient method. Finally, a jointly optimized integer matrix was formed by an iterative approach. The aforementioned studies are simulation studies or mathematical analyses. However, there are plenty of studies that have been conducted in practical experiments. A list of experimental studies is given in Table 3. The antenna configuration used by each study, modulation technique, and achieved distance are listed. From Table 3, we can observe a variety of antenna configurations and distances are used for testing MIMO VLC in experimental scenarios.

## 5. Machine Learning Based MIMO VLC

**Figure 11:**
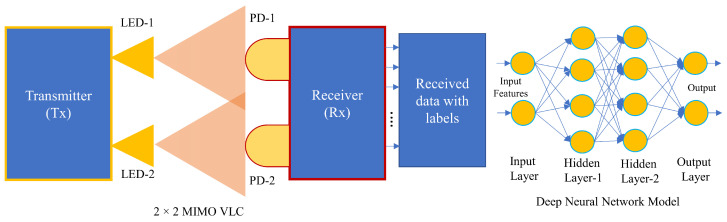
Machine Learning based VLC MIMO communication scenario.

Machine learning (ML) algorithms are emerging as an inevitable part of enhancing communication performance. Data mining, classification, prediction, and pattern recognition are all areas where ML has been successfully used [172]. To enhance the performance of signal demodulation, modulation format, and bit-rate identification, many ML techniques, including support vector machine (SVM), K-means, and density-based spatial clustering of applications with noise (DBSCAN), have been demonstrated and used [173,174,175,176,177]. By leveraging spatial diversity, optical multiple-input multiple-output (MIMO) can aid in achieving high data rates [18]. Recently, the field of VLC has been advancing MIMO technology, especially for the ML-based VLC system. The general structure of the MIMO VLC system based on ML is shown in Figure 11.

To resolve spatial multiplexing issues in VLC MIMO systems and improve spectral efficiency (SE), researchers have presented an ML-based technique called joint IQ independent component analysis (ICA) in [64]. In [64], the authors proposed a VLC based 2×2 MIMO system. The generated signals of this system are superposed signals. At the $T_{x1}$, the transmitted signals are based on 16-quadrature amplitude modulation (16-QAM), whereas at the $T_{x2}$, the transmitted signals are based on quadrature phase-shift keying (QPSK). Two optical signals can be split into two separate parallel signals using the proposed machine learning approach. MIMO-VLC receivers often utilize the MIMO decoding technique and compensator-like decision feedback equalization (DFE) to reduce spatial cross-talk and remove the inter-symbol interference step by step. Taking into account MIMO-VLC systems with inherently nonlinear nature, in [178], authors proposed an artificial neural network (ANN)-based joint spatial and temporal equalization for a MIMO-VLC system. The proposed system outperforms the joint equalization using typical decision feedback as ANN was able to reduce the non-linear transfer function as well as cross-talk using a real imaging/non-imaging optical MIMO communication channel. The joint spatial and temporal ANN equalizers were comparable to a matrix DFE. The predicted signal vector with a feedback delay line and the received signal vector with a feedforward delay line are both contained in the data structure feeding the ANN. The combination of ANN and MIMO-LMS with an adaptable parameter was proposed using two adaptive ANN (AANN) equalizers [179]. Less than 10% of MIMO-multi-branch hybrid neural network (MBNN) spatial complexity may be achieved with AANN. With carrier-less amplitude-phase (CAP) single-receiver MIMO (SR-MIMO) VLC technology and AANN equalized 16-QAM superposition coding modulation (SCM), the proposed system was able to get 2.1 Gbps data rate. To address LED non-linearity and cross-LED interference in LED MIMO communications, in [180], authors proposed extreme learning machine (ELM)-based receivers. For the proposed ELM-based receiver, a circulant input weight matrix was designed, which results in a low-complexity fast Fourier transform (FFT) implementation. In [180], the authors took into account the structure of feedforward NN with a single hidden layer, and 2×2 array LEDs were aligned with inter LEDs spacing of 0.75 m. In addition, PD was designed with an 8×8 array with spacing 0.2 m on *x* and *y*-axis, respectively. In [181], the authors proposed a deep learning network that may be used to intelligently construct MIMO-OFDM transceivers for lower symbol error probability and improved energy efficiency. To realize the signal constellation and transceivers suitable to dimmable MIMO asymmetric limiting optical-OFDM VLC systems as an end-to-end model, the concept of stacked autoencoder (SAE) was presented. The numerical outcomes demonstrate that the SAE technique outperforms the state-of-the-art zero forcing and least mean squared error algorithm in terms of bit error rate (BER) reduction. The massive MIMO-based VLC ML system was investigated in [182], where the augmented SM (ASM) was used and the complexity of ASM was examined. To demonstrate the performance, three ML model, such as SVM, logistic regression (LR), and a neural network (NN), was adopted. In this work, the identification accuracy of the transmitter, processing time, and BER were investigated. In [62], the authors proposed a hybrid ML-based VLC system that the model is called MIMO-branch hybrid neural network (MIMO-MBNN). In the single receiver-MIMO pulse amplitude magnitude eight levels VLC system, the proposed model was used as a post-equalizer. The performance comparison with others, such as single-input-single-output least mean square equalizer (SISO-LMS) and SISO deep NN showed that the proposed model gained a 3.35 dB Q factor than others. The authors in [90] proposed SVM-based detection of VLC signal in a generalized SM system, where the communication of transmitter LED and receiver PD was done by MIMO mode. The proposed system exhibited low computational complexity and optimal signal detection precision. The authors in [183] compared three MIMO schemes of RC, space-time block codes (STBCs), and SMP for indoor VLC. The results demonstrate that RC exhibits significant diversity gains as compared to the other two schemes. However, STBC and SMP can increase capacity and reliability with a slightly reduced range. Table 4 shows some of the ML-based studies, including developed models and achieved data rates.

## 6. Future Trends in VLC MIMO Communication

Different techniques and problem-solving approaches have been discussed in the previous sections. In recent years, research has been more involved in machine learning techniques and deep learning techniques. Some of the points which can be future research prospects for MIMO VLC are:

- Machine learning-based algorithms are needed to be investigated on a large scale in different MIMO scenarios.
- LOS communication is very important in VLC as the direct signal can provide high data rate communication. Reflecting intelligent surfaces [184,185] can help to reach the user with a direct signal. The channel model is very complex and needs further investigation.
- To increase connectivity in IoT device, VLC MIMO [164] can help to increase bandwidth. However, new protocols need to be investigated for margins RF and VLC to use interchangeably.
- As different levels of illumination are required in indoor environments, more efficient techniques can be investigated for dimming control without compromising data rate.
- More efficient channel estimation techniques for NLOS communication can be investigated.
- Interference is a key issue in VLC, as multiple signals can cancel out each other. Efficient power allocation in the transmitter, beamforming, or time synchronization approach can be used to investigate the reduction of interference.
- High-speed communication is still a challenge in OCC. As mobile phone is widely used, high-speed camera communication is still a challenge to overcome.
- MIMO VLC can be a research topic in implementing metaverse.
- Blockchain is a cryptocurrency system that is popular nowadays. However, the features of blockchain can be utilized in wireless networking. Research can be done to integrate blockchain into MIMO VLC.
- MIMO VLC can support the enhancement of different near-user cloud-like services like cloud computing and EDGE computing.
- As VLC can be applied in different scenarios and the number of users can be varied, different protocols can be investigated for ease of operation.

## 7. Conclusions

VLC-based communication has lots of desirable advantages which can be used to enhance future wireless communication systems. In addition, MIMO communication has played an important role in RF-based communication for a long time. Thus, MIMO VLC together can achieve the communication standard for 5G and B5G. In this paper, we have surveyed the VLC technology in the MIMO communication settings in-depth. We described VLC and the MIMO communication types available in the literature. Next, we identified different problems and categorized them, which are addressed in different studies. Machine learning approaches for MIMO VLC are also taken into account. Finally, we provided some future directions which can be investigated further.

## Figures and Tables

**Table 1:** Comparison with related studies MIMO VLC.

Ref.	MIMO Types Description	MIMO Theoretical Analysis	MIMO Experimental Analysis	Machine Learning Approaches in MIMO	Future Challenges MIMO
[13]	✓	×	×	×	✓
[40]	✓	✓	×	×	✓
[41]	✓	×	×	×	×
[42]	✓	×	×	✓	✓
[43]	✓	×	×	×	✓
This study	✓	✓	✓	✓	✓

**Table 2:** A list of studies with significant data rates for MIMO VLC.

Ref.	Antenna	Data Rate	Distance	Year	Contributions
[44]	4×9	1 Gbps	1.2 m	2013	Indoor communication with LEDs
[45]	8×8	100 Gbps	5 m	2014	Mutiuser MIMO communication with 8 channels
[46]	2×2	1.5 and 1.25 Gbps	0.75 cm	2014	imaging MIMO system with RGB LEDs
[47]	2×2	500 Mbps	40 cm	2014	non-imaging 4-QAM with Nyquist single carrier
[48]	2×2	1.8 Gbps	1.65 m	2015	equal gain combining method applied
[49]	4×4	1.2 Gbps	1 m	2015	Rectangular and linear receiver arrangement applied
[50]	2×2	1.4 Gbps	2.5 m	2016	space balance coding with RGB LEDs
[51]	3×3	1 Gbps	1 m	2016	imaging MIMO with OFDM
[52]	2×2	1 Gbps	0.6 m	2016	pre-equalizer to extend bandwidth
[53]	9×9	7.48 Gbps	0.5–1 m	2017	imaging MIMO
[54]	2×2	6.34 Gbps	1–3 m	2017	RGB-LED based wavelength division multiplexing
[55]	2×1	1.5 Gbps	1.3 m	2018	detection algorithm using the successive interference cancellation (SIC) and the look-up table
[56]	4×4	249 Mbps	4.5 m	2018	multi-band carrierless amplitude and phase modulation
[57]	2×2	1.6 Gbps	1 m	2019	BER improvement
[58]	2×2	5 Gbps	2 m	2019	64QAM-DMT modulation
[59]	4×4	2.3–1.7 Gbps	1–4 m	2019	color-polarization multiplexing method
[60]	4×4	1 Gbps	indoor	2019	Multi-color MIMO VLC
[61]	14,400 × 400	4 Gbps	2 m	2020	Massive MIMO using space division multiple access for supporting multiple users
[62]	2×1	2.1 Gbps	1.2 m	2020	single receiver MIMO VLC with neural network
[63]	2×2	1.8484 Gbps	up to 5 m	2020	Probabilistic shaping bitloading MIMO
[64]	2×2	750 Mbps	1.3 m	2020	machine learning based MIMO detection scheme
[65]	2×2	3.08 Gbps, 336 Mbps (daytime) and 362 Mbps (nighttime)	2 m and 100 m	2021	MIMO vehicular communication using VLC
[66]	2×4	5.4 Gbps	1.5 m	2022	CAP-16 QAM system based on a Si-substrate golden light LED array

**Table 3:** Experimental Study on MIMO VLC Communication.

Ref.	Antenna	Modulation	Distance
[152]	2 × 1	COOK	20 m
[157]	4 × 4	OFDM	0.1 m
[50]	2 × 2	QAM-OFDM	2.5 m
[158]	2 × 2	OOK	2 m
[118]	2 × 2	4-QAM	0.35 m
[56]	4 × 4	M-QAM	2.5 m
[159]	2 × 2 and 4 × 4	PPM, OOK, PWM & MPPM	1–21 m
[44]	4 × 9	OFDM	1 m
[160]	2 × 2	OOK	0.1m
[130]	2 × 2 and 2 × 1	OOK	6–14 m
[118]	2 × 2	NOMA (QAM)	0.15–0.35 m
[161]	3 × 3	DCO-OFDM	0.1 m
[94]	2 × 2	4-QAM and 8-QAM	1.1 m
[162]	3 × 3	4-QAM and 2-PSK	20 m
[163]	3 × 3	WDM	2 m
[164]	2 × 2	OOK and MPPM	15 m
[56]	4 × 4	M-QAM	4.5 m
[165]	2 × 2	OOK	6 m
[166]	2 × 2	OFDM	0.8 m
[167]	4 × 4	OOK	10 m
[168]	3 × 3	OFDM	1 m
[51]	3 × 3	OFDM	1 m
[58]	2 × 2	64-QAM	2 m
[169]	4 × 6	TDMA and SDMA	variable distance
[114]	4 × 4	2-PAM	3 m
[149]	8 × 6	16-QAM	5 m
[170]	3 × 3	OOK-NRZ	0.75 m
[171]	2 × 2	OOK	0.25 m
[64]	2 × 2	QPSK and 16-QAM	1.3 m
[66]	2 × 2	16-QAM	1.5 m
[148]	2 × 2	BPSK	1.2 m

**Table 4:** Overview of ML based MIMO VLC Communication.

Ref.	System	ML Model	Distance	Achievable Rate
[64]	2 × 2	joint IQ ICA	1.3 m	750 Mbps
[180]	2 × 2 LED, 8 × 8 PD	ELM-NN	1.75 m	-
[181]	4 × 4	SAE-ANN	2.65 m	92.31 Mbps to 676.26 Mbps
[178]	2 × 2	ANN	1 m	1975 Mbps
[62]	2 × 2	MBNN	1.2 m	2.1 Gbps
[179]	2 × 2	AANN	1.2 m	2.1 Gbps
[90]	4 × 4, 8 × 4	SVM	2.15 m	-

## Data Availability

Not applicable.

## References

[1] Chen X., Ng D.W.K., Yu W., Larsson E.G., Al-Dhahir N., Schober R. (2021). Massive Access for 5G and Beyond. IEEE J. Sel. Areas Commun..

[2] Gui G., Liu M., Tang F., Kato N., Adachi F. (2020). 6G: Opening new horizons for integration of comfort, security, and intelligence. IEEE Wirel. Commun..

[3] Miramirkhani F., Karbalayghareh M., Zeydan E., Mitra R. (2022). Enabling 5G indoor services for residential environment using VLC technology. Phys. Commun..

[4] Cisco U. (2020). Cisco Annual Internet Report (2018–2023) White Paper.

[5] An J., Sejan M.A.S., Chung W.Y. (2019). Fine particulate matter monitoring via a visible light communication in DCT-based optical OFDM. Opt. Express.

[6] Sejan M.A.S., Naik R.P., Lee B.G., Chung W.Y. (2022). A Bandwidth Efficient Hybrid Multilevel Pulse Width Modulation for Visible Light Communication System: Experimental and Theoretical Evaluation. IEEE Open J. Commun. Soc..

[7] Khan L.U. (2017). Visible light communication: Applications, architecture, standardization and research challenges. Digit. Commun. Netw..

[8] Rahaim M.B., Vegni A.M., Little T.D. A hybrid radio frequency and broadcast visible light communication system. Proceedings of the 2011 IEEE GLOBECOM Workshops (GC Wkshps).

[9] Gismalla M.S., Abdullah M.F.L., Ahmed M.S., Mabrouk W.A., Najib A.F., Saeid E., Supa’at A., Das B. (2021). Design and analysis of different optical attocells deployment models for indoor visible light communication system. Int. J. Integr. Eng..

[10] Rahaim M.B., Little T.D. (2015). Toward practical integration of dual-use VLC within 5G networks. IEEE Wirel. Commun..

[11] Jovicic A., Li J., Richardson T. (2013). Visible light communication: Opportunities, challenges and the path to market. IEEE Commun. Mag..

[12] Rahman M.H., Sejan M.A.S., Chung W.Y. (2021). Multilateration Approach for Wide Range Visible Light Indoor Positioning System Using Mobile CMOS Image Sensor. Appl. Sci..

[13] Pathak P.H., Feng X., Hu P., Mohapatra P. (2015). Visible Light Communication, Networking, and Sensing: A Survey, Potential and Challenges. IEEE Commun. Surv. Tutor..

[14] Matheus L.E.M., Vieira A.B., Vieira L.F.M., Vieira M.A.M., Gnawali O. (2019). Visible Light Communication: Concepts, Applications and Challenges. IEEE Commun. Surv. Tutor..

[15] Sejan M.A.S., Chung W.Y. (2020). Indoor fine particulate matter monitoring in a large area using bidirectional multihop VLC. IEEE Internet Things J..

[16] Duman T.M., Ghrayeb A. (2008). Coding for MIMO Communication Systems.

[17] Larsson E.G., Edfors O., Tufvesson F., Marzetta T.L. (2014). Massive MIMO for next generation wireless systems. IEEE Commun. Mag..

[18] Mesleh R., Mehmood R., Elgala H., Haas H. Indoor MIMO optical wireless communication using spatial modulation. Proceedings of the 2010 IEEE International Conference on Communications.

[19] Merdan F.A.B., Thiagarajah S.P., Dambul K. (2022). Non-line of sight visible light communications: A technical and application based survey. Optik.

[20] Xie Y., Ho I.W.H., Magsino E.R. (2017). The modeling and cross-layer optimization of 802.11 p VANET unicast. IEEE Access.

[21] Ingason T., Liu H. (2009). Line-of-Sight MIMO for Microwave Links-Adaptive Dual Polarized and Spatially Separated Systems. Master’s Thesis.

[22] Furukawa H., Kamio Y., Sasaoka H. Co-channel interference reduction method using CMA adaptive array antenna. Proceedings of the PIMRC’96-7th International Symposium on Personal, Indoor, and Mobile Communications.

[23] Hosney M., Selmy H.A., Srivastava A., Elsayed K.M. (2020). Interference mitigation using angular diversity receiver with efficient channel estimation in MIMO VLC. IEEE Access.

[24] Sevincer A., Bhattarai A., Bilgi M., Yuksel M., Pala N. (2013). LIGHTNETs: Smart LIGHTing and mobile optical wireless NETworks—A survey. IEEE Commun. Surv. Tutor..

[25] Karunatilaka D., Zafar F., Kalavally V., Parthiban R. (2015). LED based indoor visible light communications: State of the art. IEEE Commun. Surv. Tutor..

[26] Ergul O., Dinc E., Akan O.B. (2015). Communicate to illuminate: State-of-the-art and research challenges for visible light communications. Phys. Commun..

[27] Rahman M.H., Sejan M.A.S., Kim J.J., Chung W.Y. (2020). Reduced tilting effect of smartphone CMOS image sensor in visible light indoor positioning. Electronics.

[28] Rahman M.H., Sejan M.A.S. Performance Analysis of Indoor Positioning System Using Visible Light Based on Two-LEDs and Image Sensor for Different Handhold Situation of Mobile Phone. Proceedings of the 2020 IEEE Region 10 Symposium (TENSYMP).

[29] Do T.H., Yoo M. (2016). An in-depth survey of visible light communication based positioning systems. Sensors.

[30] Căilean A.M., Dimian M. (2016). Toward environmental-adaptive visible light communications receivers for automotive applications: A review. IEEE Sensors J..

[31] Căilean A.M., Dimian M. (2017). Current Challenges for Visible Light Communications Usage in Vehicle Applications: A Survey. IEEE Commun. Surv. Tutor..

[32] Luo J., Fan L., Li H. (2017). Indoor positioning systems based on visible light communication: State of the art. IEEE Commun. Surv. Tutor..

[33] Rehman S.U., Ullah S., Chong P.H.J., Yongchareon S., Komosny D. (2019). Visible light communication: A system perspective—Overview and challenges. Sensors.

[34] Blinowski G. (2019). Security of visible light communication systems—A survey. Phys. Commun..

[35] Mapunda G.A., Ramogomana R., Marata L., Basutli B., Khan A.S., Chuma J.M. (2020). Indoor visible light communication: A tutorial and survey. Wirel. Commun. Mob. Comput..

[36] Yahia S., Meraihi Y., Ramdane-Cherif A., Gabis A.B., Acheli D., Guan H. (2021). A survey of channel modeling techniques for visible light communications. J. Netw. Comput. Appl..

[37] Oyewobi S.S., Djouani K., Kurien A.M. (2022). Visible Light Communications for Internet of Things: Prospects and Approaches, Challenges, Solutions and Future Directions. Technologies.

[38] Geng Z., Khan F.N., Guan X., Dong Y. (2022). Advances in Visible Light Communication Technologies and Applications. Photonics.

[39] Vappangi S., Mani V. (2022). A survey on the integration of visible light communication with power line communication: Conception, applications and research challenges. Optik.

[40] Ishikawa N., Sugiura S., Hanzo L. (2018). 50 years of permutation, spatial and index modulation: From classic RF to visible light communications and data storage. IEEE Commun. Surv. Tutor..

[41] Chataut R., Akl R. (2020). Massive MIMO systems for 5G and beyond networks—overview, recent trends, challenges, and future research direction. Sensors.

[42] Al-Ahmadi S., Maraqa O., Uysal M., Sait S.M. (2018). Multi-user visible light communications: State-of-the-art and future directions. IEEE Access.

[43] Vishwaraj, Ali L. Hybrid MIMO-OFDM system for 5G network using VLC—A review. Proceedings of the 2019 IEEE International Conference on Electrical, Computer and Communication Technologies (ICECCT).

[44] Azhar A.H., Tran T.A., O’Brien D. (2012). A gigabit/s indoor wireless transmission using MIMO-OFDM visible-light communications. IEEE Photonics Technol. Lett..

[45] Chang C.H., Li C.Y., Lu H.H., Lin C.Y., Chen J.H., Wan Z.W., Cheng C.J. (2014). A 100-Gb/s multiple-input multiple-output visible laser light communication system. J. Light. Technol..

[46] Wang Y., Chi N. (2014). Indoor gigabit 2 ë 2 imaging multiple-input-multiple-output visible light communication. Chin. Opt. Lett..

[47] Wang Y., Chi N. (2014). Demonstration of high-speed 2 × 2 non-imaging MIMO Nyquist single carrier visible light communication with frequency domain equalization. J. Light. Technol..

[48] Shi J., Huang X., Wang Y., Tao L., Chi N. Improved performance of a high speed 2 × 2 MIMO VLC network based on EGC-STBC. Proceedings of the 2015 European Conference on Optical Communication (ECOC).

[49] Zhan Z., Zhang M., Han D., Luo P., Tang X., Ghassemlooy Z., Lang L. 1.2 Gbps non-imaging MIMO-OFDM scheme based VLC over indoor lighting LED arrangments. Proceedings of the 2015 Opto-Electronics and Communications Conference (OECC).

[50] Li J., Xu Y., Shi J., Wang Y., Ji X., Ou H., Chi N. (2016). A 2 × 2 imaging MIMO system based on LED visible light communications employing space balanced coding and integrated PIN array reception. Opt. Commun..

[51] Hsu C.W., Chow C.W., Lu I.C., Liu Y.L., Yeh C.H., Liu Y. (2016). High speed imaging 3 × 3 MIMO phosphor white-light LED based visible light communication system. IEEE Photonics J..

[52] Lu I.C., Liu Y.L., Lai C.H. High-speed 2 × 2 MIMO-OFDM visible light communication employing phosphorescent LED. Proceedings of the 2016 Eighth International Conference on Ubiquitous and Future Networks (ICUFN).

[53] Rajbhandari S., Jalajakumari A.V., Chun H., Faulkner G., Cameron K., Henderson R., Tsonev D., Haas H., Xie E., McKendry J.J. (2017). A multigigabit per second integrated multiple-input multiple-output VLC demonstrator. J. Light. Technol..

[54] Lu I.C., Lai C.H., Yeh C.H., Chen J. 6.36 Gbit/s RGB LED-based WDM MIMO visible light communication system employing OFDM modulation. Proceedings of the 2017 Optical Fiber Communication Conference.

[55] Qiao L., Lu X., Liang S., Zhang J., Chi N. (2018). Performance analysis of space multiplexing by superposed signal in multi-dimensional VLC system. Opt. Express.

[56] Werfli K., Chvojka P., Ghassemlooy Z., Hassan N.B., Zvanovec S., Burton A., Haigh P.A., Bhatnagar M.R. (2018). Experimental demonstration of high-speed 4 × 4 imaging multi-CAP MIMO visible light communications. J. Light. Technol..

[57] Hong Y., Chen L.K., Zhao J. (2019). Performance-enhanced gigabit/s MIMO-OFDM visible light communications using CSI-free/dependent precoding techniques. Opt. Express.

[58] Shi M., Wang C., Li G., Liu Y., Wang K., Chi N. A 5Gb/s 2 × 2 MIMO Real-time Visible Light Communication System based on silicon substrate LEDs. Proceedings of the 2019 Global LIFI Congress (GLC).

[59] Yeh C.H., Weng J.H., Chow C.W., Luo C.M., Xie Y.R., Chen C.J., Wu M.C. (2019). 1.7 to 2.3 Gbps OOK LED VLC transmission based on 4 × 4 color-polarization-multiplexing at extremely low illumination. IEEE Photonics J..

[60] Xiao Y., Zhu Y., Zhang D., Zhang H. High-Speed visible light communication chipset based multi-color MIMO system. Proceedings of the 2019 28th Wireless and Optical Communications Conference (WOCC).

[61] Younus S.H., Al-Hameed A.A., Alhartomi M., Hussein A.T. Massive MIMO For Indoor VLC Systems. Proceedings of the 2020 22nd International Conference on Transparent Optical Networks (ICTON).

[62] Zou P., Zhao Y., Hu F., Chi N. (2020). Enhanced performance of MIMO multi-branch hybrid neural network in single receiver MIMO visible light communication system. Opt. Express.

[63] Jia J., Zou P., Hu F., Zhao Y., Chi N. (2020). Flexible Data Rate V2X Communication System beyond 1.84 Gb/s Based on MIMO VLC and Radar Integration. Appl. Sci..

[64] Wang Z., Han S., Chi N. (2020). Performance enhancement based on machine learning scheme for space multiplexing 2 × 2 MIMO VLC system employing joint IQ independent component analysis. Opt. Commun..

[65] Li G., Niu W., Ha Y., Hu F., Wang J., Yu X., Jia J., Zou P., He Z., Yu S. (2022). Position-Dependent MIMO Demultiplexing Strategy for High-Speed Visible Light Communication in Internet of Vehicles. IEEE Internet Things J..

[66] Niu W., Xu Z., Xiao W., Liu Y., Hu F., Wang G., Zhang J., He Z., Yu S., Shi J. (2022). Phosphor-free Golden Light LED Array for 5.4-Gbps Visible Light Communication using MIMO Tomlinson-Harashima Precoding. J. Light. Technol..

[67] Sejan M.A.S., Chung W.Y. (2022). Secure VLC for Wide-Area Indoor IoT Connectivity. IEEE Internet Things J..

[68] Sejan M.A.S., Chung W.Y. (2020). Lightweight multi-hop VLC using compression and data-dependent multiple pulse modulation. Opt. Express.

[69] Islim M.S., Haas H. (2019). Modulation techniques for li-fi. ZTE Commun..

[70] Sejan M.A.S., Rahman M.H., Chung W.Y. MPPM based bi-directional long range visible light communication for indoor particulate matter monitoring. Proceedings of the 2020 IEEE 3rd International Conference on Computer and Communication Engineering Technology (CCET).

[71] Komine T., Nakagawa M. (2004). Fundamental analysis for visible-light communication system using LED lights. IEEE Trans. Consum. Electron..

[72] Goldsmith A. (2005). Wireless Communications.

[73] Zeng L., O’Brien D.C., Le Minh H., Faulkner G.E., Lee K., Jung D., Oh Y., Won E.T. (2009). High data rate multiple input multiple output (MIMO) optical wireless communications using white LED lighting. IEEE J. Sel. Areas Commun..

[74] Chi N. (2018). LED-Based Visible Light Communications.

[75] Sejan M.A.S., Rahman M.H., Chung W.Y. Optical OFDM Modulation in Multi-hop VLC for Long Distance Data Transmission Over 30 meters. Proceedings of the 2020 IEEE Photonics Conference (IPC).

[76] Fath T., Haas H. (2012). Performance comparison of MIMO techniques for optical wireless communications in indoor environments. IEEE Trans. Commun..

[77] Safari M., Uysal M. (2008). Do we really need OSTBCs for free-space optical communication with direct detection?. IEEE Trans. Wirel. Commun..

[78] Dixit V., Kumar A. (2021). Performance analysis of angular diversity receiver based MIMO–VLC system for imperfect CSI. J. Opt..

[79] Siddiqi U.F., Narmanlioglu O., Uysal M., Sait S.M. (2020). Joint bit and power loading for adaptive MIMO OFDM VLC systems. Trans. Emerg. Telecommun. Technol..

[80] Tran N.A., Luong D.A., Thang T.C., Pham A.T. Performance analysis of indoor MIMO visible light communication systems. Proceedings of the 2014 IEEE Fifth International Conference on Communications and Electronics (ICCE).

[81] Chau Y.A., Yu S.H. Space modulation on wireless fading channels. Proceedings of the IEEE 54th Vehicular Technology Conference. VTC Fall 2001. Proceedings (Cat. No. 01CH37211).

[82] Mesleh R.Y., Haas H., Sinanovic S., Ahn C.W., Yun S. (2008). Spatial modulation. IEEE Trans. Veh. Technol..

[83] Mesleh R., Elgala H., Haas H. (2011). Optical spatial modulation. J. Opt. Commun. Netw..

[84] Yang P., Xiao Y., Yu Y., Li S. (2011). Adaptive spatial modulation for wireless MIMO transmission systems. IEEE Commun. Lett..

[85] Xiao Y., Zhu Y.J. (2019). Chromaticity-adaptive generalized spatial modulation for MIMO VLC with multi-color LEDs. IEEE Photonics J..

[86] Wang J.Y., Ge H., Zhu J.X., Wang J.B., Dai J., Lin M. (2018). Adaptive spatial modulation for visible light communications with an arbitrary number of transmitters. IEEE Access.

[87] Younis A., Serafimovski N., Mesleh R., Haas H. Generalised spatial modulation. Proceedings of the 2010 confterence Record of the Forty Fourth Asilomar Conference on Signals, Systems and Computers.

[88] Alaka S., Narasimhan T.L., Chockalingam A. Generalized spatial modulation in indoor wireless visible light communication. Proceedings of the 2015 IEEE Global Communications Conference (GLOBECOM).

[89] Kumar C.R., Jeyachitra R. (2017). Power efficient generalized spatial modulation MIMO for indoor visible light communications. IEEE Photonics Technol. Lett..

[90] Sun H., Zhang Y., Wang F., Zhang J., Shi S. (2021). SVM Aided Signal Detection in Generalized Spatial Modulation VLC System. IEEE Access.

[91] Bolcskei H. (2006). MIMO-OFDM wireless systems: Basics, perspectives, and challenges. IEEE Wirel. Commun..

[92] He C., Wang T.Q., Armstrong J. Performance comparison between spatial multiplexing and spatial modulation in indoor MIMO visible light communication systems. Proceedings of the 2016 IEEE International Conference on Communications (ICC).

[93] Butala P.M., Elgala H., Little T.D. Performance of optical spatial modulation and spatial multiplexing with imaging receiver. Proceedings of the 2014 IEEE Wireless Communications and Networking Conference (WCNC).

[94] Guo X., Chi N. (2019). Superposed 32QAM constellation design for 2 × 2 spatial multiplexing MIMO VLC systems. J. Light. Technol..

[95] Zhai Y., Chi H., Tong J., Xi J. (2020). Capacity maximized linear precoder design for spatial-multiplexing MIMO VLC systems. IEEE Access.

[96] Guo X., Yuan Y., Pan C., Xiao J. (2021). Interleaved superposed-64QAM-constellation design for spatial multiplexing visible light communication systems. Opt. Express.

[97] Ying K., Qian H., Baxley R.J., Yao S. (2015). Joint optimization of precoder and equalizer in MIMO VLC systems. IEEE J. Sel. Areas Commun..

[98] Yang H., Chen C., Zhong W.D., Alphones A. (2018). Joint precoder and equalizer design for multi-user multi-cell MIMO VLC systems. IEEE Trans. Veh. Technol..

[99] Zhai Y., Tong J., Xi J. (2019). Precoder design for MIMO visible light communications with decision-feedback receivers. IEEE Photonics Technol. Lett..

[100] Subramani P., Rajendran G.B., Sengupta J., Pérez de Prado R., Divakarachari P.B. (2020). A block bi-diagonalization-based pre-coding for indoor multiple-input-multiple-output-visible light communication system. Energies.

[101] Wu L., Cheng J., Zhang Z., Dang J., Liu H. (2017). Channel estimation for optical-OFDM-based multiuser MISO visible light communication. IEEE Photonics Technol. Lett..

[102] He X., Song R., Zhu W.P. (2013). Pilot allocation for sparse channel estimation in MIMO-OFDM systems. IEEE Trans. Circuits Syst. II Express Briefs.

[103] Lin B., Ghassemlooy Z., Xu J., Lai Q., Shen X., Tang X. (2020). Experimental demonstration of compressive sensing-based channel estimation for MIMO-OFDM VLC. IEEE Wirel. Commun. Lett..

[104] Ashok D., Chockalingam A. Compact optimal pilot design for channel estimation in MIMO VLC systems. Proceedings of the 2019 IEEE Wireless Communications and Networking Conference (WCNC).

[105] Alamir A., Esmaiel H., Hussein H.S. (2020). Efficient Optical MIMO–OFDM Channel Estimation Based on Correntropy Compressive Sensing. Wirel. Pers. Commun..

[106] Hong Y., Chen J., Wang Z., Yu C. (2013). Performance of a precoding MIMO system for decentralized multiuser indoor visible light communications. IEEE Photonics J..

[107] Wang Q., Wang Z., Dai L. (2015). Multiuser MIMO-OFDM for visible light communications. IEEE Photonics J..

[108] Narmanlioglu O., Uysal M. Limited feedback channel estimation for multi-user massive mimo visible light communications. Proceedings of the ICC 2020-2020 IEEE International Conference on Communications (ICC).

[109] Chen C., Zhang R., Wen W., Liu M., Du P., Yang Y., Ruan X. (2022). Hybrid 3DMA for multi-user MIMO-VLC. J. Opt. Commun. Netw..

[110] Cai K., Jiang M. Multi-user MIMO-OOFDM imaging VLC system with PD selection. Proceedings of the 2016 IEEE 83rd Vehicular Technology Conference (VTC Spring).

[111] Beysens J., Galisteo A., Wang Q., Juara D., Giustiniano D., Pollin S. DenseVLC: A cell-free massive MIMO system with distributed LEDs. Proceedings of the 14th International Conference on Emerging Networking EXperiments and Technologies.

[112] Arfaoui M.A., Ghrayeb A., Assi C.M. (2018). Secrecy performance of multi-user MISO VLC broadcast channels with confidential messages. IEEE Trans. Wirel. Commun..

[113] Burton A., Ghassemlooy Z., Rajbhandari S., Liaw S.K. (2014). Design and analysis of an angular-segmented full-mobility visible light communications receiver. Trans. Emerg. Telecommun. Technol..

[114] Nuwanpriya A., Ho S.W., Chen C.S. (2015). Indoor MIMO Visible Light Communications: Novel Angle Diversity Receivers for Mobile Users. IEEE J. Sel. Areas Commun..

[115] Fahamuel P., Thompson J., Haas H. Improved indoor VLC MIMO channel capacity using mobile receiver with angular diversity detectors. Proceedings of the 2014 IEEE Global Communications Conference.

[116] Rahman M.H., Sejan M.A.S., Yoo S.G., Kim M.A., You Y.H., Song H.K. (2022). Multi-User Joint Detection Using Bi-Directional Deep Neural Network Framework in NOMA-OFDM System. Sensors.

[117] Dai L., Wang B., Yuan Y., Han S., Chih-Lin I., Wang Z. (2015). Non-orthogonal multiple access for 5G: Solutions, challenges, opportunities, and future research trends. IEEE Commun. Mag..

[118] Lin B., Ghassemlooy Z., Tang X., Li Y., Zhang M. (2017). Experimental demonstration of optical MIMO NOMA-VLC with single carrier transmission. Opt. Commun..

[119] Shi J., Hong Y., He J., Deng R., Chen L.K. Experimental demonstration of OQAM-OFDM based MIMO-NOMA over visible light communications. Proceedings of the 2018 Optical Fiber Communication Conference. Optical Society of America.

[120] Wang H., Wang F., Li R. (2020). Enhancing power allocation efficiency of NOMA aided-MIMO downlink VLC networks. Opt. Commun..

[121] Liu X., Yu H., Zhu Y., Zhang E. Power allocation algorithm of optical MIMO NOMA visible light communications. Proceedings of the 2019 IEEE 9th International Conference on Electronics Information and Emergency Communication (ICEIEC).

[122] Mishra A.K., Trivedi A. Performance Analysis of MIMO-NOMA-Based Indoor Visible Light Communication in Single Reflection Environment. Proceedings of the 2019 IEEE Conference on Information and Communication Technology.

[123] Raj R., Dixit A. Performance evaluation of power allocation schemes for non-orthogonal multiple access in MIMO visible light communication links. Proceedings of the 2020 International Conference on Signal Processing and Communications (SPCOM).

[124] Jha M.K., Kumar N., Lakshmi Y. NOMA MIMO Visible Light Communication with ZF-SIC and MMSE-SIC. Proceedings of the 2020 2nd PhD Colloquium on Ethically Driven Innovation and Technology for Society (PhD EDITS).

[125] Rajput V.S., Ashok D., Chockalingam A. MU-MIMO NOMA with linear precoding techniques in indoor downlink VLC systems. Proceedings of the 2020 IEEE 91st Vehicular Technology Conference (VTC2020-Spring).

[126] Dixit V., Kumar A. (2022). Error analysis of L-PPM modulated MIMO based multi-user NOMA-VLC system with perfect and imperfect SIC. Appl. Opt..

[127] Nguyen T., Islam A., Hossan T., Jang Y.M. (2017). Current status and performance analysis of optical camera communication technologies for 5G networks. IEEE Access.

[128] Rahman M.H., Sejan M.A.S., Chung W.Y. Long-Distance Real-Time Rolling Shutter Optical Camera Communication Using MFSK Modulation Technique. Proceedings of the International Conference on Intelligent Human Computer Interaction.

[129] Chen S.H., Chow C.W. (2015). Hierarchical scheme for detecting the rotating MIMO transmission of the in-door RGB-LED visible light wireless communications using mobile-phone camera. Opt. Commun..

[130] Liu Y., He W. Signal detection and identification in an optical camera communication system in moving state. Proceedings of the 2021 2nd International Workshop on Electronic communication and Artificial Intelligence (IWECAI 2021).

[131] Han B., Hranilovic S. (2017). A fixed-scale pixelated MIMO visible light communication system. IEEE J. Sel. Areas Commun..

[132] Teli S.R., Matus V., Zvanovec S., Perez-Jimenez R., Vitek S., Ghassemlooy Z. The first study of mimo scheme within rolling-shutter based optical camera communications. Proceedings of the 2020 12th International Symposium on Communication Systems, Networks and Digital Signal Processing (CSNDSP).

[133] Zhu Y.J., Liang W.F., Zhang J.K., Zhang Y.Y. (2015). Space-collaborative constellation designs for MIMO indoor visible light communications. IEEE Photonics Technol. Lett..

[134] Guo X., Yuan Y., Zhao Y., Chi N. (2022). Flipped superposed constellation design for MIMO visible-light communication systems. Opt. Express.

[135] Sun X., Kang C.H., Kong M., Alkhazragi O., Guo Y., Ouhssain M., Weng Y., Jones B.H., Ng T.K., Ooi B.S. (2020). A review on practical considerations and solutions in underwater wireless optical communication. J. Light. Technol..

[136] Song Y., Lu W., Sun B., Hong Y., Qu F., Han J., Zhang W., Xu J. (2017). Experimental demonstration of MIMO-OFDM underwater wireless optical communication. Opt. Commun..

[137] Yilmaz A., Elamassie M., Uysal M. Diversity gain analysis of underwater vertical MIMO VLC links in the presence of turbulence. Proceedings of the 2019 IEEE International Black Sea Conference on Communications and Networking (BlackSeaCom).

[138] Li Y., Qiu H., Chen X., Fu J., Musa M., Li X. (2019). Spatial correlation analysis of imaging MIMO for underwater visible light communication. Opt. Commun..

[139] AL-Deen M.B., Ali M.A.A., Saleh Z.A. (2021). Improving the optical link for UVLC using MIMO technique. J. Opt. Commun..

[140] Jamali M.V., Nabavi P., Salehi J.A. (2018). MIMO underwater visible light communications: Comprehensive channel study, performance analysis, and multiple-symbol detection. IEEE Trans. Veh. Technol..

[141] Mumtaz S., Aziz A.A., Masroor K. On the Performance of MIMO-UVLC System over Turbulence-induced Fading Channels. Proceedings of the 2022 International Conference on Artificial Intelligence for Smart Community.

[142] Meucci M., Seminara M., Nawaz T., Caputo S., Mucchi L., Catani J. (2022). Bidirectional Vehicle-to-Vehicle Communication System Based on VLC: Outdoor Tests and Performance Analysis. IEEE Trans. Intell. Transp. Syst..

[143] Narmanlioglu O., Turan B., Ergen S.C., Uysal M. (2018). Cooperative MIMO-OFDM based inter-vehicular visible light communication using brake lights. Comput. Commun..

[144] Petrariu A.I., Lavric A., Coca E. VLC for vehicular communications: A multiple input multiple output (MIMO) approach. Proceedings of the 2018 International Conference on Development and Application Systems (DAS).

[145] Yahia S., Meraihi Y., Refas S., Gabis A.B., Ramdane-Cherif A., Eldeeb H.B. (2022). Performance study and analysis of MIMO visible light communication-based V2V systems. Opt. Quantum Electron..

[146] Wang T.Q., Sekercioglu Y.A., Armstrong J. (2013). Analysis of an optical wireless receiver using a hemispherical lens with application in MIMO visible light communications. J. Light. Technol..

[147] Kumar A., Ghorai S. (2018). Effect of multipath reflection on BER performance of indoor MIMO-VLC system. Opt. Quantum Electron..

[148] Chen C., Yang Y., Deng X., Du P., Yang H. (2020). Space division multiple access with distributed user grouping for multi-user MIMO-VLC systems. IEEE Open J. Commun. Soc..

[149] Berenguer P.W., Schulz D., Hilt J., Hellwig P., Kleinpeter G., Fischer J.K., Jungnickel V. (2017). Optical wireless MIMO experiments in an industrial environment. IEEE J. Sel. Areas Commun..

[150] Bober K.L., Mana S.M., Hinrichs M., Kouhini S.M., Kottke C., Schulz D., Schmidt C., Freund R., Jungnickel V. (2021). Distributed multiuser mimo for lifi in industrial wireless applications. J. Light. Technol..

[151] Butala P.M., Elgala H., Little T.D. SVD-VLC: A novel capacity maximizing VLC MIMO system architecture under illumination constraints. Proceedings of the 2013 IEEE Globecom Workshops (GC Wkshps).

[152] Nguyen V.H., Thieu M.D., Nguyen H., Jang Y.M. (2020). Design and Implementation of the MIMO–COOK Scheme Using an Image Sensor for Long-Range Communication. Sensors.

[153] Le Tran M., Kim S. (2019). Effective Receiver Design for MIMO Visible Light Communication with Quadrichromatic LEDs. Electronics.

[154] Hussein H.S., Hagag M., Farrag M. (2020). Extended spatial-index LED modulation for optical MIMO-OFDM wireless communication. Electronics.

[155] Bai R., Jang R., Tan J., Quan J. Performance comparison of VLC MIMO techniques considering indoor illuminance with inclined LEDs. Proceedings of the 2016 IEEE International Conference on Wireless for Space and Extreme Environments (WiSEE).

[156] Huang N., Wang X., Chen M. (2017). Transceiver design for MIMO VLC systems with integer-forcing receivers. IEEE J. Sel. Areas Commun..

[157] Wei L., Zhang H., Song J. (2016). Experimental demonstration of a cubic-receiver-based MIMO visible light communication system. IEEE Photonics J..

[158] Burton A., Le Minh H., Ghassemlooy Z., Bentley E., Botella C. (2014). Experimental Demonstration of 50-Mb/s Visible Light Communications Using 4x4 MIMO. IEEE Photonics Technol. Lett..

[159] Sejan M.A.S., Chung W.Y. (2022). Performance Analysis of a Long-Range MIMO VLC System for Indoor IoT. IEEE Internet Things J..

[160] Li S., Huang B., Xu Z. Experimental MIMO VLC systems using tricolor LED transmitters and receivers. Proceedings of the 2017 IEEE Globecom Workshops (GC Wkshps).

[161] Jung H., Kim S.M. (2021). Experimental Demonstration of 3 × 3 MIMO LED-to-LED Communication Using RGB Colors. Sensors.

[162] Turan B., Narmanlioglu O., Ergen S.C., Uysal M. Broadcasting brake lights with MIMO-OFDM based vehicular VLC. Proceedings of the 2016 IEEE Vehicular Networking Conference (VNC).

[163] Omura N., Higashi A., Yabuuchi J., Iwamatsu T., Oshiba S. Experimental demonstration of ofdm based wdm-mimo visible light communication system. Proceedings of the 2018 Asia-Pacific Microwave Conference (APMC).

[164] Sejan M.A.S., Chung W.Y. MIMO Based Indoor Visible Light Communication for Internet of Things via Data Encryption. Proceedings of the 2021 IEEE 7th World Forum on Internet of Things (WF-IoT).

[165] Jesuthasan F.H., Rohitkumar H., Shah P., Trestian R. Implementation and performance evaluation of a MIMO-VLC system for data transmissions. Proceedings of the 2019 IEEE International Symposium on Broadband Multimedia Systems and Broadcasting (BMSB).

[166] Guo X., Wang C., Wang W. (2020). Experimental Demonstration of Zadoff–Chu Matrix Transform Precoding for MIMO-OFDM Visible Light Communications. Adv. Condens. Matter Phys..

[167] Kim S.M., Jeon J.B. (2012). Experimental demonstration of 4 × 4 MIMO wireless visible light communication using a commercial CCD image sensor. J. Inf. Commun. Converg. Eng..

[168] Hsu C.W., Lu I.C., Liu Y.L., Yeh C.H., Chow C.W. Demonstration of high speed imaging 3×3 MIMO-OFDM visible light communication system. Proceedings of the 2016 IEEE Photonics Conference (IPC).

[169] Mana S.M., Jungnickel V., Bober K.L., Hellwig P., Hilt J., Schulz D., Paraskevopoulos A., Freund R., Hirmanova K., Janca R. (2021). Distributed Multiuser MIMO for LiFi: Experiments in an Operating Room. J. Light. Technol..

[170] Burton A., Chvojka P., Haigh P.A., Ghassemlooy Z., Zvanovec S. (2021). Optical Filter-Less WDM for Visible Light Communications Using Defocused MIMO. Electronics.

[171] Kowalczyk M. (2016). 2 × 2 MIMO VLC optical transmission system based on LEDs in a double role. Acta Phys. Pol. A.

[172] Chi N., Zhao Y., Shi M., Zou P., Lu X. (2018). Gaussian kernel-aided deep neural network equalizer utilized in underwater PAM8 visible light communication system. Opt. Express.

[173] Lu X., Qiao L., Zhou Y., Yu W., Chi N. (2019). An IQ-Time 3-dimensional post-equalization algorithm based on DBSCAN of machine learning in CAP VLC system. Opt. Commun..

[174] Lu X., Zhou Y., Qiao L., Yu W., Liang S., Zhao M., Zhao Y., Lu C., Chi N. (2019). Amplitude jitter compensation of PAM-8 VLC system employing time-amplitude two-dimensional re-estimation base on density clustering of machine learning. Phys. Scr..

[175] Qiao L., Liang S., Lu X., Chi N. (2019). Distortion compensation with clustering algorithm in a MISO VLC system. Opt. Commun..

[176] Niu W., Ha Y., Chi N. Novel phase estimation scheme based on support vector machine for multiband-CAP visible light communication system. Proceedings of the Asia Communications and Photonics Conference. Optica Publishing Group.

[177] Wu X., Chi N. (2019). The phase estimation of geometric shaping 8-QAM modulations based on K-means clustering in underwater visible light communication. Opt. Commun..

[178] Rajbhandari S., Chun H., Faulkner G., Haas H., Xie E., McKendry J.J., Herrnsdorf J., Gu E., Dawson M.D., O’Brien D. (2019). Neural network-based joint spatial and temporal equalization for MIMO-VLC system. IEEE Photonics Technol. Lett..

[179] Zhao Y., Zou P., He Z., Li Z., Chi N. (2021). Low spatial complexity adaptive artificial neural network post-equalization algorithms in MIMO visible light communication systems. Opt. Express.

[180] Gao D., Guo Q. (2019). Extreme learning machine-based receiver for MIMO LED communications. Digit. Signal Process..

[181] Hao L., Li C., Wang D. (2021). Quantitative analysis of the stacked autoencoder method in MIMO-ACO-OFDM VLC systems. Light. Res. Technol..

[182] Khadr M.H., Walter I., Elgala H., Muhaidat S. (2020). Machine learning-based massive augmented spatial modulation (ASM) for IoT VLC systems. IEEE Commun. Lett..

[183] Khalid A., Asif H.M., Mumtaz S., Al Otaibi S., Konstantin K. (2019). Design of MIMO-visible light communication transceiver using maximum rank distance codes. IEEE Access.

[184] Sejan M.A.S., Rahman M.H., Shin B.S., Oh J.H., You Y.H., Song H.K. (2022). Machine Learning for Intelligent-Reflecting-Surface-Based Wireless Communication towards 6G: A Review. Sensors.

[185] Sejan M.A.S., Rahman M.H., Song H.K. (2022). Demod-CNN: A Robust Deep Learning Approach for Intelligent Reflecting Surface-Assisted Multiuser MIMO Communication. Sensors.

